# Mining and functional characterization of NADPH-cytochrome P450 reductases of the DNJ biosynthetic pathway in mulberry leaves

**DOI:** 10.1186/s12870-024-04815-0

**Published:** 2024-02-23

**Authors:** Yangzhen Liao, Wenmin Du, Jingqiong Wan, Jiahe Fan, Jilan Pi, Min Wu, Yuan Wei, Zhen Ouyang

**Affiliations:** 1 School of Food and Biological Engineering, Jiangsu University, Zhenjiang, 212013 PR China; 2 School of Pharmacy, Jiangsu University, Zhenjiang, 212013 PR China

**Keywords:** *Morus alba* L., NADPH-cytochrome P450 reductases, Functional characterization

## Abstract

**Background:**

1-Deoxynojirimycin (DNJ), the main active ingredient in mulberry leaves, with wide applications in the medicine and food industries due to its significant functions in lowering blood sugar, and lipids, and combating viral infections. Cytochrome P450 is a key enzyme for DNJ biosynthesis, its activity depends on the electron supply of NADPH-cytochrome P450 reductases (CPRs). However, the gene for MaCPRs in mulberry leaves remains unknown.

**Results:**

In this study, we successfully cloned and functionally characterized two key genes, *MaCPR1* and *MaCPR2*, based on the transcriptional profile of mulberry leaves. The *MaCPR1* gene comprised 2064 bp, with its open reading frame (ORF) encoding 687 amino acids. The *MaCPR2* gene comprised 2148 bp, and its ORF encoding 715 amino acids. The phylogenetic tree indicates that MaCPR1 and MaCPR2 belong to Class I and Class II, respectively. In vitro, we found that the recombinant enzymes MaCPR2 protein could reduce cytochrome *c* and ferricyanide using NADPH as an electron donor, while MaCPR1 did not. In yeast, heterologous co-expression indicates that MaCPR2 delivers electrons to MaC3'H hydroxylase, a key enzyme catalyzing the production of chlorogenic acid from 3-O-p-coumaroylquinic acid.

**Conclusions:**

These findings highlight the orchestration of hydroxylation process mediated by MaCPR2 during the biosynthesis of secondary metabolite biosynthesis in mulberry leaves. These results provided a foundational understanding for fully elucidating the DNJ biosynthetic pathway within mulberry leaves.

**Supplementary Information:**

The online version contains supplementary material available at 10.1186/s12870-024-04815-0.

## Introduction

Mulberry leaves, derived from the mulberry plant (*Morus alba* L.), are highly valuable as both medicinal and food resources, which were globally cultivated and have been used for the prevention and treatment of diabetes since ancient times. 1-deoxynojirimycin (DNJ), a polyhydroxy alkaloid component, was considered the main active ingredient in mulberry leaves [1, 2]. Studies have found that DNJ, as an effective α-glucosidase inhibitor, could significantly inhibit the increase in blood sugar, lower blood lipids and reduce atherosclerosis, and also has anti-oxidant, anti-virus and anti-tumor, etc. [3–5]. In addition, the unique physiological and pharmacological properties of DNJ allow it to show great potential in the creation of new drugs against diabetes and antiviral. However, the natural abundance of DNJ is very low, its main natural source of mulberry leaves only ~ 0.1% DNJ [6, 7], but the high separation costs and low resource utilization. At the same time, chemical synthesis and microbial transformation encounter challenges such as isomer separation difficulty and low yield [8, 9]. This significantly limits the utilization of polyhydroxy alkaloids DNJ in the pharmaceutical, food and other fields. Consequently, finding innovative and efficient ways to obtain DNJ alkaloids has emerged as an urgent challenge. Biosynthesis as an alternative method, is environmentally friendly and is expected to be a novel way to efficiently obtain DNJ alkaloid [10]. However, the key enzyme genes in its biosynthetic pathway remain incompletely understood.

DNJ belongs to the piperidine class of polyhydroxy alkaloids, with a hydroxymethyl group at the C2-position and a hydroxyl group at the C3-, C4- and C5-positions of the piperidine ring, respectively. The polyhydroxy structure endows it with various pharmacological activity [11]. There are two speculations about the biosynthesis pathway of DNJ in mulberry leaves [12–14]. Theory of biosynthesis mechanism based on secondary metabolites of alkaloids in plants. DNJ is a piperidine polyhydroxy alkaloid originating from lysine [15, 16], which undergoes a series of biochemical reactions by functional enzymes such as decarboxylation, oxidation, and reduction to form piperidines, as well as finally generates DNJ under the action of cytochrome P450 and methyltransferases [17]. Another DNJ biosynthetic pathway is derived from glucose, which undergoes a series of biochemical reactions such as reduction, oxidation, dehydration, and isomerization in the presence of functional enzymes to form DNJ [9, 13]. The biosynthetic pathways of secondary metabolites in plants are very complex, and multiple biosynthetic pathways may have existed during evolution due to biotic or abiotic stresses [10]. Therefore, both hypothesized DNJ biosynthetic pathways may be present in mulberry leaves, but either biosynthetic pathway requires the involvement of an electron supply system. We previously investigated the biosynthetic pathway by which DNJ originates from lysine. We cloned and characterized of lysine decarboxylase (MaLDC, MG727866) [12], copper amine oxidase (MaCAO, MH205733) [18], reductases (MaSDRs, MT989445, MT989446) [19], and methyltransferases (MaMTs, OM140666) [17] by comparative transcriptomics and metabolomics approaches, and preliminary elucidation of the lysine to 2-methylpiperidine biosynthetic pathway. It is hypothesized that, the downstream pathway is cytochrome P450 enzymes (CYP450s) mediated hydroxylation, ultimately leading to the formation of DNJ. The stereoselective hydroxylation of these CYP450s determines the diversity of hydroxyl structures of DNJ, as well as its derivatives [20]. The catalytic activity of CYP450s relies on their companion cytochrome P450 reductase (CPR, E.C.1.6.2.4) [21]. CPR utilizes FAD as the electron transfer port, accepting a pair of electrons from electron donor NADPH and converting them into hydride ions. Subsequently, it utilizes FMN as the electron outgoing port, facilitating the transfer of electrons to the iron ion center on the iron porphyrin molecule of CYP450. This process supplies electrons for a series of oxidation reactions [22, 23]. Therefore, MaCPRs play an indispensable role in the post-modification of the DNJ biosynthetic pathway.

The electron transfer reaction between CPR and CYP is a crucial step in the CYP-catalyzed oxidation reaction, pairing CYP with a suitable CPR might potentially improve the catalytic efficiency of the CYP450 system [21]. The fusion expression or co-expression of CPR with CYP450 in yeast is a common method to characterize the effect of CPR on CYP450 catalysis [24]. For example, the screening of novel-found CYP450 and CPR genes greatly increased the yield of glycyrrhizic acid and 11-O-β-amyrin in the biosynthetic pathways of *Saccharomyces cerevisiae* (*S. cerevisiae*) [25]. Both PtCPR1 and PtCPR2 from *Hybrid poplar* presented to support the catalytic activity of cinnamate-4-hydroxylase (C4H) by yeast co-expression [26]. However, there have not been any reports about MaCPRs in mulberry leaves. In this study, we cloned and bioinformatically analyzed two *MaCPRs* genes screened from the transcriptional profile of mulberry leaves, characterizing their functions by prokaryotic and eukaryotic expressions. The results of this study potentially contribute to the elucidation of hydroxylation process within DNJ biosynthetic pathway of mulberry leaves, also providing references for the study of secondary metabolites in mulberry leaves. The research provides selectable genetic resources for the construction of microbial expression systems and large-scale industrial production of DNJ.

## Materials and methods

### RNA isolation and cDNA synthesis

We harvested fresh and young mulberry leaves from the Mulberry Garden of Jiangsu University, Zhenjiang, China and were identified as leaves of *Morus alba* L. by Professor Zhen Ouyang. Three well-grown mulberry trees were selected and the first to third tender leaf at the top were collected every ten days from July 15 to November 15, 2022 at 9:00 a.m. These leaves were quickly frozen with liquid nitrogen, and stored at -80 °C. To extract RNA, we used Beyozol reagent (Beyotime Biotechnology, Shanghai, China), and subsequently converted it into cDNA using a first-strand cDNA synthesis kit (Thermo Fisher Scientific, Waltham, MA, USA). cDNA was stored at -20 °C for later use.

### Cloning and bioinformatics analysis of *MaCPR1* and *MaCPR2*

The truncated *MaCPR1* and *MaCPR2* genes were amplified by PCR from the transcriptional profile of mulberry leaves (Supplementary tables S4, S5, S6**)**. The PCR products were ligated to the pMD18-T vector (TaKaRa Biotechnology, Dalian, China), and then transformed towards *Escherichia coli* DH5α (Sangon Biotechnology, Shanghai, China). Positive colonies were screened by plates containing ampicillin for PCR verification, and confirmed by bidirectional sequencing. Nucleotide and conserved domain analysis were performed by National Center for Biotechnology Information (NCBI). The physicochemical properties analysis of MaCPR1 and MaCPR2 gene encoding protein by ExPASy ProtParam tool (http://web.expasy.org/protparam/), and analyzed the presence of signal peptides and transmembrane helices by SignalP 5.0 Server (http://www.cbs.dtu.dk/services/SignalP/) and TMHMM (http://www.cbs.dtu.dk/services/TMHMM-2.0/), and predicted the secondary and 3D structures by SOPMA SECONDARY STRUCTURE PREDICTION METHOD (https://npsa-prabi.ibcp.fr/cgi-bin/npsa_automat.pl?page=npsa_sopma.html) and AlphaFold2.0 (https://colab.research.google.com/github/sokrypton/ColabFold/blob/main/AlphaFold2.ipynb). The phylogenetic trees and multiple sequence alignments were constructed by MEGA 7.0 software based on the protein sequences. The Neighbor-Joining (NJ) method was selected for tree-building, and bootstrapping was conducted with 1000 cycles to test the confidence of each branch.

### Prokaryotic expression and protein purification of MaCPR1 and MaCPR2

The cloned MaCPR1 and MaCPR2 genes were ligated with the prokaryotic expression vector pET-32a( +) (Invitrogen Corporation, Carlsbad, USA), subsequently transformed towards *E. coli* BL21 (DE3) (Sangon Biotechnology, Shanghai, China), confirmed by bidirectional sequencing. The correctly sequenced bacteria were incubated to an OD_600_ = 0.6 ~ 0.8, and then isopropyl-*β*-D-thiogalactopyranoside (IPTG) (Sangon Biotechnology, Shanghai, China) was added to the Luria–Bertani (LB) medium until consequent concentration (1 mM) for induce protein expression for 16 h at 16℃ and 200 rpm. The precipitate was collected by centrifugation (4℃, 9000 × *g*), added non-denaturing lysate (50 mM Tris, 500 mM NaCl, PH = 7.5), and then added protease inhibitor phenylmethylsulfonyl fluoride (PMSF) to the final concentration of 100 μg/mL. The cells were ultrasonically crushed on ice for 10 min (ultrasonic power of 400 W, working for 5 s and intermittent for 5 s). Subsequently, the resultant supernatant was obtained via centrifugation (4℃, 9000 × *g*). MaCPR1 and MaCPR2 proteins were purified using the Denaturant-resistant His-tag Purification Resin kit (Sangon Biotechnology, Shanghai, China) following professional protocols. The purity of the protein was verified by SDS-PAGE, and the quantification of protein concentration was accomplished by BCA Protein Assay Kit (Sangon Biotechnology, Shanghai, China).

### In vitro enzyme activity assays of MaCPR1 and MaCPR2

In vitro enzyme activity analysis of recombinant proteins MaCPR1 and MaCPR2 was performed with minor modifications to previous methods [27]. To investigate the electron transfer activity of recombinant MaCPR1 and MaCPR2 to cytochrome *c*. The reactions happened in 200 μL volume containing 20 μg MaCPR1 or MaCPR2 recombinant proteins, NADPH (100 μM), Tris–HCl buffer (50 mM, pH = 7.4, 0.1 mM EDTA), and cytochrome *c* (ranging from 0 to 200 μM) at 25℃, and was detected at OD_550_ using microplate reader. The determination method of NADPH kinetic parameters is as follows, the reactions were conducted in a 200 μL mixture containing 20 μg MaCPR1 or MaCPR2 recombinant proteins, cytochrome *c* (200 μM), Tris–HCl buffer (50 mM, pH = 7.4, 0.1 mM EDTA), and NADPH (ranging from 0 to 200 μM) at 25℃, and was detected at OD_550_ through microplate reader. To investigate the electron transfer activity of MaCPR1 and MaCPR2 to K_3_Fe(CN)_6_. The following methods were adopted: the reactions in 200 μL mixture containing 20 μg of MaCPR1 or MaCPR2 recombinant proteins, NADPH (100 μM), Tris–HCl buffer (50 mM, pH = 7.4, 0.1 mM EDTA), and K_3_Fe(CN)_6_ (ranging from 0 to 200 μM) at 25℃, and was detected at OD_424_ a using microplate reader. Origin software was adopted to draw the protein activity curve. The data presented is based on three independent experiments.

### Heterologous co-expression of MaCPR2 and MaC3'H in yeast

The functional characterization of CYP450 involved in the DNJ biosynthetic pathway is still under investigation. The coumaroyl ester 3'-hydroxylase (MaC3'H) hydroxylase has been verified to have a hydroxylation function in mulberry leaves [28, 29], and was used to verify the function of MaCPRs as CPR. The *MaC3'H* and *MaCPR2* genes were ligated into the promoter pESC-Trp expression frames of GAL1 and GAL10 using T4 DNA ligase, respectively, thus the co-expression expression vector MaC3'H/pESC-Trp and MaC3'H-MaCPR2/pESC-Trp was constructed. All eukaryotic expression vectors were confirmed by restriction endonuclease and bidirectional sequencing. The empty vectors pESC-Trp, MaC3'H/pESC-Trp, and MaC3'H-MaCPR2/pESC-Trp were transformed into yeast INVSCI competent cells, using Frozen-EZ Yeast Transformation II kit (ZYMO Research, Los Angeles, USA). The single clone was inoculated in 1 mL SD liquid medium without tryptophan at a shaking table at 30 °C. The total culture volume was expanded to 10 mL with a 5% inoculation amount, and the yeast cells were collected by centrifugation. Then, the yeast cells were rinsed with sterilized water and repeated 3 times to remove the residual glucose. Added 10 mL of SG liquid medium without tryptophan to yeast cells, after 10 h of incubation at 30 °C, 3-O-p-coumaroylquinic acid was added to the yeast culture to a final concentration of 100 μM, and the yeast cells were cultured for another 48 h. The products of each reaction were extracted with 2 volumes of methanol. The resultant supernatant was harvested by centrifugation (12,000 × *g*, 5 min) and spun-dried by a rotary evaporator, subsequently dissolved in 800 µL of methanol. High-performance liquid chromatography (HPLC) and mass spectrometry (MS) were performed through a 0.22 µm filter membrane.

### Product identification by LC–MS/MS

The Kromasil C_18_ column was chosen for HPLC (Agilent Technology, Palo Alto, USA). The mobile phases were acetonitrile (A) and 0.2% acetic acid aqueous solution (B) [29]. Gradient elution was adopted, and the gradient was: 0–5 min, 10–90%; 5–50 min, 52–48%; 50–55 min, 10–90%. The flow rate was set at 0.8 mL/min. Injection volume was 20 μL. Column temperature was set at 30 °C. Detection wavelength was 327 nm. The conditions of mass spectrometry were electrospray ionization source (ESI) of positive and negative ion modes were used for analysis. Spray voltage was set to 3.5 kV. Interface temperature was set to 300 °C. Temperature of desolventizing was 526 °C. Flow rate of heating gas was 10 L/min. Flow rate of atomizing gas was set to 3 L/min. Scanning range is 50–500 m/z.

## Results

### Screening of *MaCPRs* candidate genes from mulberry leaves

To investigate the biosynthetic pathway of DNJ in mulberry leaves, the transcriptome sequencing of mulberry leaves (Ma_7 Vs Ma_11) was performed and its transcriptome data were obtained (SRA, Accession NO. SRP127713). Eleven gene sequences annotated as MaCPRs were obtained from the transcriptome profiles of mulberry leaves. To ensure the integrity of the proteins encoded by the gene sequences, we screened the sequences based on their length and found that only two MaCPRs genes had more complete coding functional regions. They were named *MaCPR1* (Unigene ID: c42829_g1) and *MaCPR2* (Unigene ID: c44129_g1), the length of open reading frame (ORF) is 2064 bp and 2148 bp, respectively. The ORF sequences of the two genes were compared with other species' information in the database of NCBI. The results illustrated that *MaCPR1* and *MaCPR2* genes had more than 80% identity with CPR sequences of *Morus notabilis*, *Cannabis sativa*, *Prunus mume* and other species. Therefore, *MaCPR1* and *MaCPR2* genes in transcriptome profiles are used as candidate genes for further research.

### Cloning and bioinformatics analysis of *MaCPR1* and *MaCPR2*

To obtain the soluble proteins of MaCPR1 and MaCPR2. Primers were designed by truncating 2 ~ 61 amino acids at the N-terminus of MaCPR1 protein and 2 ~ 82 amino acids at the N-terminus of MaCPR2 protein, respectively. All primer designs are shown in Table 1. The truncated *MaCPR1* (1881 bp) and *MaCPR2* (1902 bp) genes were cloned from mulberry leaves (Supplementary figure S1). Sequencing results affirmed their congruence with the sequences of c42829_g1 and c44129_g1 in transcriptome data. The complete ORF size of MaCPR1 spans 2064 bp, encoding 687 amino acids, with a predicted molecular weight (Mw) of 76.6 kDa and an isoelectric point (pI) of 5.25. Similarly, the complete ORF size of MaCPR2 is 2148 bp, encoding 715 amino acids, with a predicted Mw of 79.0 kDa and pI of 5.17. Signal peptide prediction results indicated the absence of a signal peptide in the predicted peptide, suggesting that MaCPR1 and MaCPR2 proteins are non-excretory proteins (Supplementary figure S3). The results predicted by TMHMM showed that the transmembrane domain of MaCPR1 protein was between the 13 and 35 amino acids, and MaCPR2 protein was between the 54 and 73 amino acids (Supplementary figure S4). The secondary structure prediction showed that the proteins encoded by MaCPR1 and MaCPR2 contain 41.78% and 42.38% α-helix, 14.85% and 13.85% extension chain, 4.37% and 3.78% β-turn, 39.01% and 40.00% random curl, respectively (Supplementary figure S5). The prediction of tertiary structures of proteins encoded by MaCPR1 and MaCPR2 genes by AlphaFold 2.0. The PLDDT values were 87.7 for MaCPR1 and 85.2 for MaCPR2, indicating that the three-dimensional (3D) modeling of MaCPR1 and MaCPR2 proteins was of good quality and the results had high confidence (Supplementary figure S6).

**Table 1:** List of primers used in this study

Gene names	Primer sequences (5′ → 3′)	Application
*MaCPR1-* Forward	CGGGATCCATGCTGGTCAAGGACGAAG	CDS cloning
*MaCPR1—*Reverse	GCGTCGACCCAGACATCTCTCAGATATCGTC	CDS cloning
*MaCPR2-* Forward	CCCAAGCTTGGATGAAGGCGGTGGAGCTTCTC	CDS cloning
*MaCPR2—*Reverse	GCCCTCGAGCCACACATCACGTAGATACCTGCC	CDS cloning
*MaCPR2-* Forward-Trp	ATTTGCGGCCGCATGGACTCCTCGTCGTC	Yeast expression
*MaCPR2—*Reverse-Trp	ATTTGCGGCCGCTCACCACACATCACGTAGAT	Yeast expression
*MaC3'H*—Forward-Trp	CGGGATCCATGGATCTCCTTCTAATCATTCC	Yeast expression
*MaC3'H—*Reverse-Trp	CCCTGAGTTACACATCCACAACCTCGC	Yeast expression

**Fig. 1:**
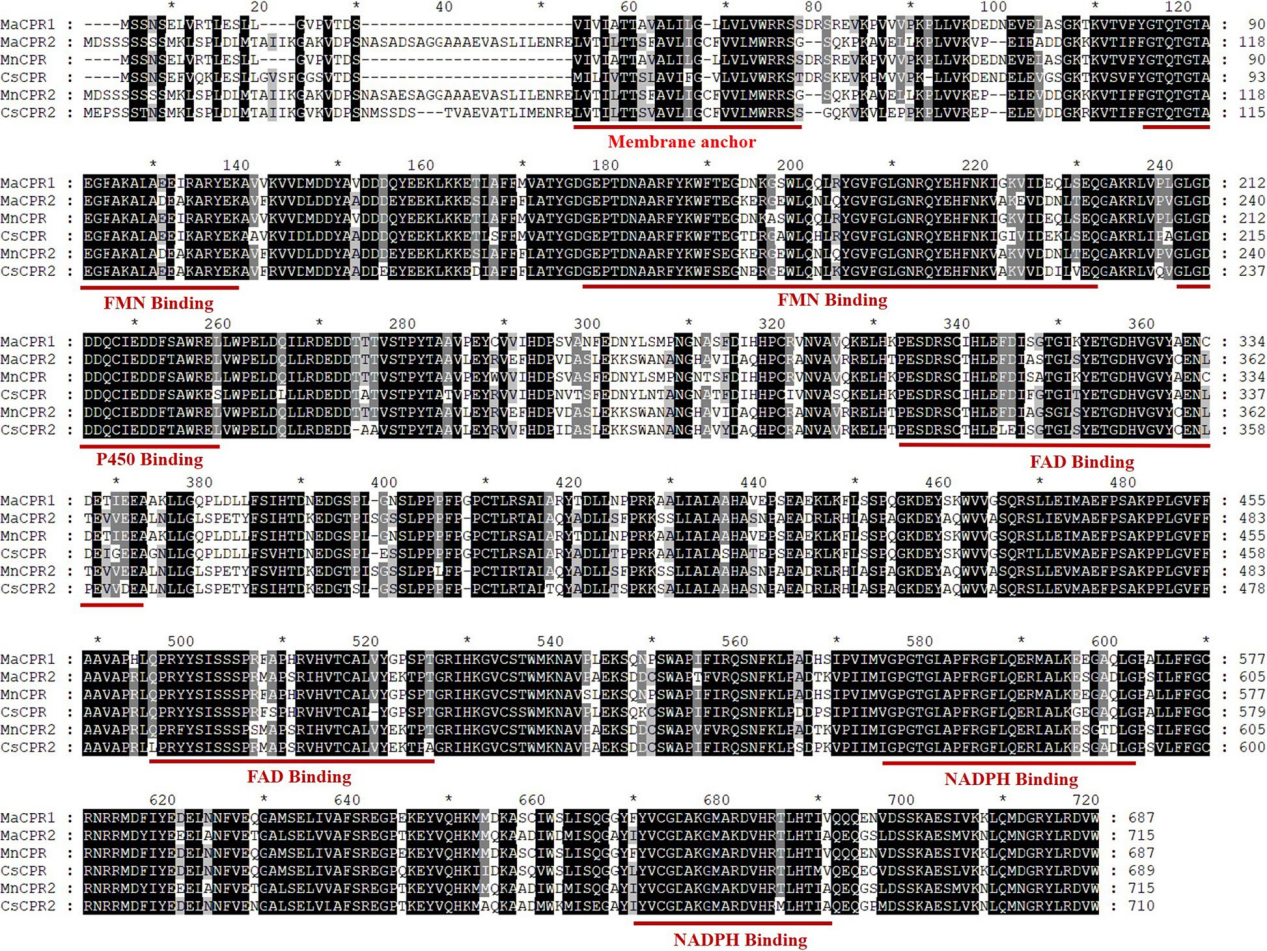
Multiple sequence alignment of MaCPR1 and MaCPR2 with CPR of other species. MaCPR1 (*Morus alba*), MaCPR2 (*Morus alba*), MnCPR (*Morus notabilis*, XP-010086660.1), CSCPR (*Cannabis sativa*, XP-030489299.1), MnCPR2 (*Morus notabilis*, XP_010093854.1), CSCPR2 (*Cannabis sativa*, XP_030477763.1)

**Fig. 2:**
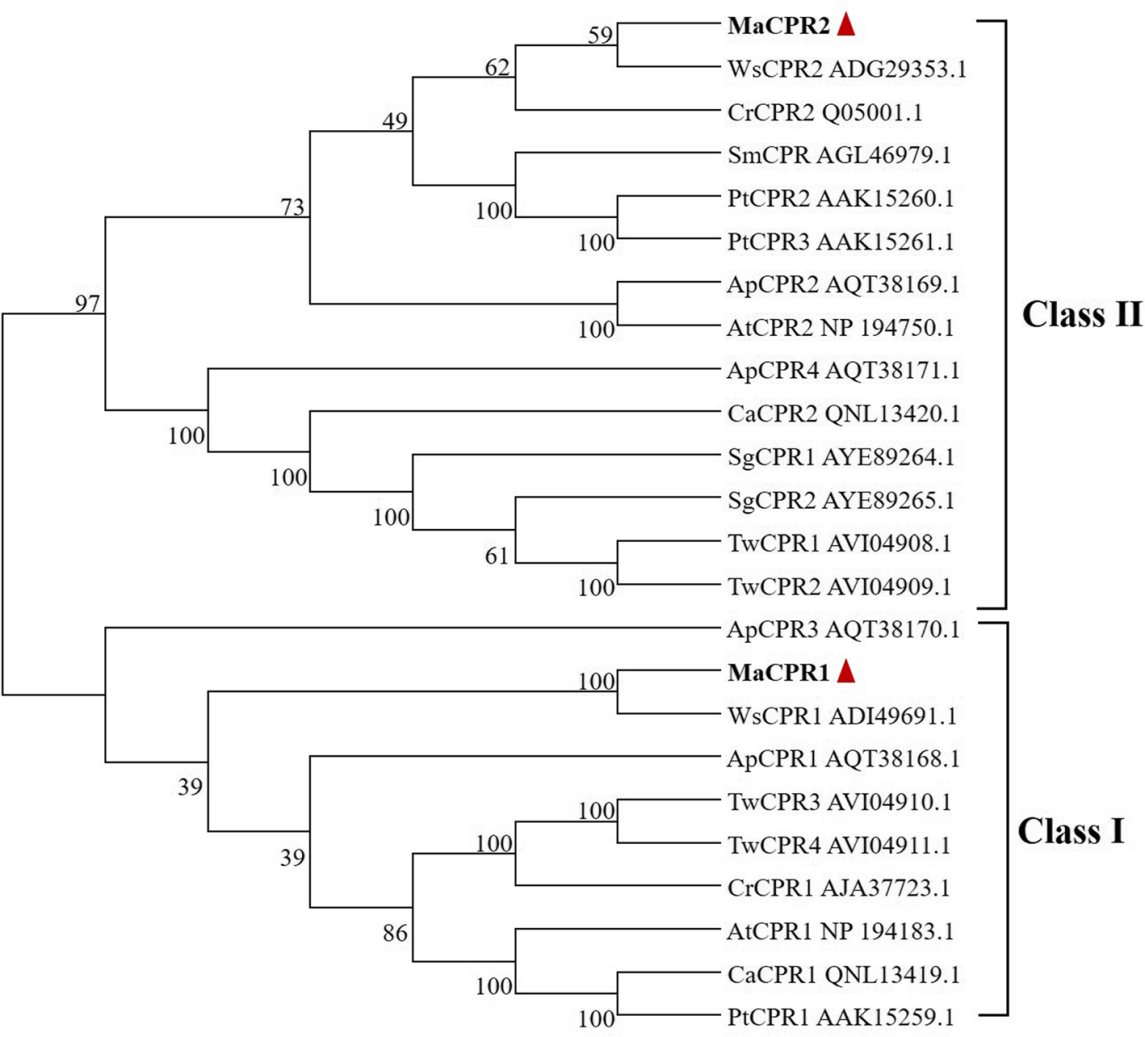
The phylogenetic trees of MaCPR1 and MaCPR2 were constructed by MEGA 7.0 software based on the protein sequences. The Neighbor-Joining (NJ) method was selected for tree-building, and bootstrapping was conducted with 1000 cycles to test the confidence of each branch. MaCPR2 (*Morus alba*); WsCPR2 (*Withania somnifera*, ADG29353.1); CrCPR2 (*Catharanthus roseus*, Q05001.1); SmCPR (*Salvia miltiorrhiza*, AGL46979.1); PtCPR2 (*Populus trichocarpa x Populus deltoides*, AAK15260.1); PtCPR3 (*Populus trichocarpa x Populus deltoides*, AAK15261.1); ApCPR2 (*Andrographis paniculate*, AQT38169.1); AtCPR2 (*Arabidopsis thaliana*, NP_194750.1); ApCPR4 (*Andrographis paniculate*, AQT38171.1); CaCPR2 (*Camptotheca acuminata*, QNL13420.1); SgCPR1 (*Siraitia grosvenorii*, AYE89264.1); SgCPR2 (*Siraitia grosvenorii*, AYE89265.1); TwCPR1 (*Tripterygium wilfordii*, AVI04908.1); TwCPR2 (*Tripterygium wilfordii*, AVI04909.1); MaCPR1 (*Morus alba*); WsCPR1 (*Withania somnifera*, ADI49691.1); ApCPR1 (*Andrographis paniculate*, AQT38168.1); TwCPR3 (*Tripterygium wilfordii*, AVI04910.1); TwCPR4 (*Tripterygium wilfordii*, AVI04911.1); CrCPR1 (*Catharanthus roseus*, AJA37723.1); AtCPR1 (*Arabidopsis thaliana*, NP_194183.1); CaCPR1 (*Camptotheca acuminata*, QNL13419.1); PtCPR1(*Populus trichocarpa x Populus deltoide,* AAK15259.1)

The outcomes of multiple sequence alignments revealed that the anticipated amino acid sequences of MaCPR1 and MaCPR2 exhibit conserved eukaryotic cytochrome P450 reductase domains. These encompass the N-terminal membrane anchoring/FMN binding/P450 binding/FAD-binding/NADPH binding domains (Fig. 1), the length of N-terminal of MaCPR1 is shorter than MaCPR2. The phylogenetic trees results showed that MaCPR1 was the closest relative to CPR1 proteins in *Withania somnifera*, clustered with CPR1 in *Arabidopsis thaliana*, MaCPR2 showed the closest relation to CPR2 proteins within *Withania somnifera*, clustered with CPR2 in *Arabidopsis thaliana* (Fig. 2). Currently, CPR proteins in plants are classified into two main groups, whereas MaCPR1 belongs to Class I, while MaCPR2 to Class II, which is consistent with the results of multiple sequence comparisons.

### Prokaryotic expression and purification of recombinant proteins MaCPR1 and MaCPR2

**Fig. 3:**
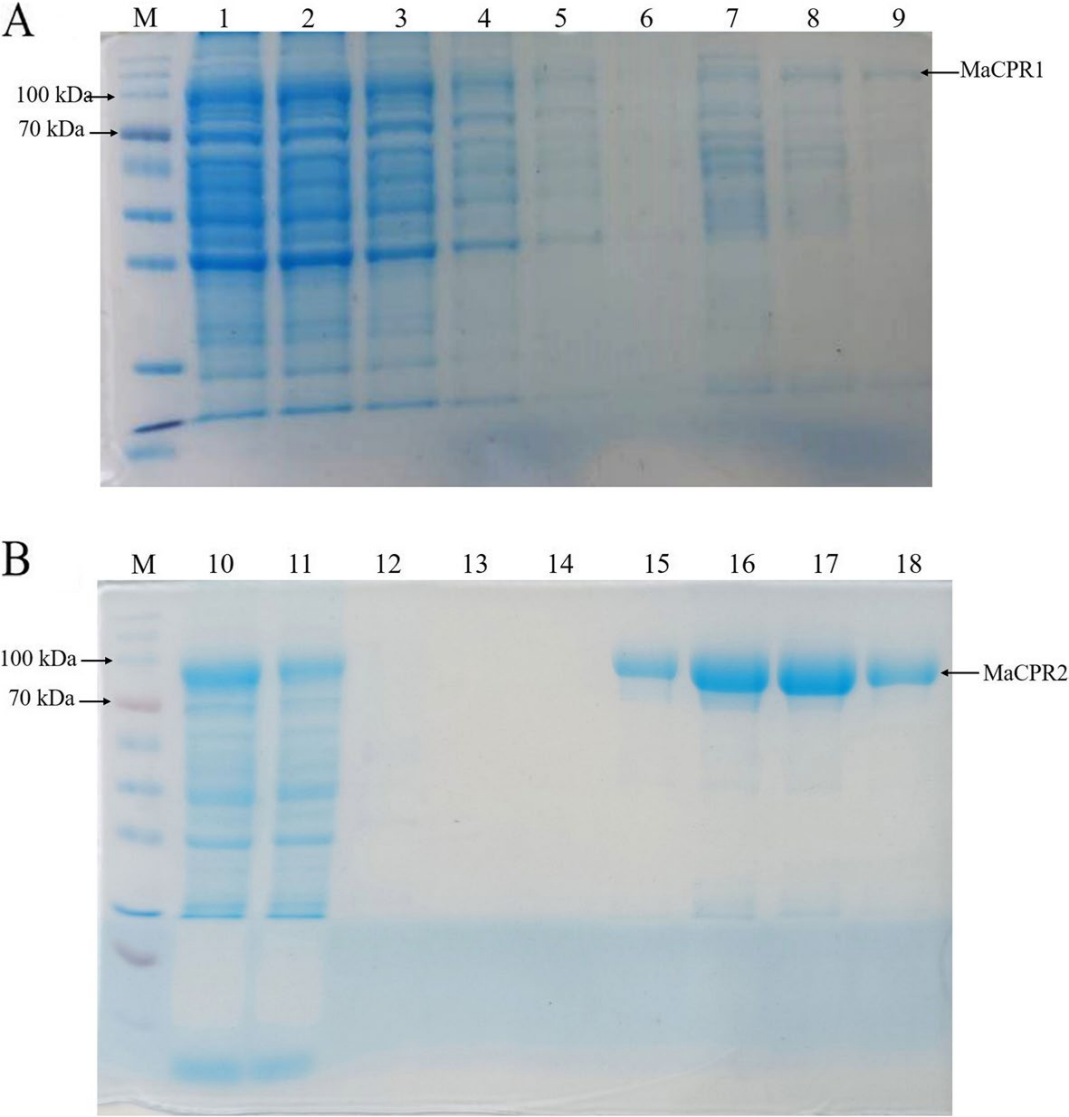
SDS-PAGE analysises of purified recombinant proteins MaCPR1 and MaCPR2. **A** SDS-PAGE analysises of purified recombinant proteins MaCPR1, Lane M: Protein molecular markers (10–170 kDa); Lane 1: Supernatant after lysis of *E. coli* BL21 (DE3); Lane 2: Flow through liquid; Lane 3–9: Imidazole eluent at different concentrations. **B** SDS-PAGE analysises of purified recombinant proteins MaCPR2, Lane M: Protein molecular markers (10–170 kDa); Lane 10: Supernatant after lysis of *E. coli* BL21 (DE3); Lane 11: Flow through liquid; Lane 12–18: Imidazole eluent at different concentrations

The previously cloned truncated gene sequences of MaCPR1 and MaCPR2 were ligated into the pET-32a( +) expression vector, incorporating a fused His-tag. The recombinant proteins, MaCPR1 and MaCPR2, were discernibly expressed in the supernatant of *E. coli* BL21 (DE3) cell lysate. Subsequent purification of the recombinant proteins revealed, through SDS-PAGE analysis, that MaCPR1 exhibited bands within the range of 70 kDa to 100 kDa. The recombinant MaCPR1 protein displayed an estimated size of approximately 90.75 kDa, including the His-tag (Fig. 3A, Supplementary figure S7A, 10A). Similarly, MaCPR2 exhibited bands spanning 70 kDa to 100 kDa, with the recombinant MaCPR2 protein manifesting an estimated size of about 91.33 kDa, encompassing the His-tag (Fig. 3B, Supplementary figures S7B, 10B). Subsequent analysis of BCA protein concentration based on a standard curve indicated that the purified protein concentrations of recombinant MaCPR1 and MaCPR2 were 0.257 and 2.3 mg/mL, respectively (Supplementary figure S8).

### Determination of enzyme activities of recombinant proteins MaCPR1 and MaCPR2 in vitro

**Fig. 4:**
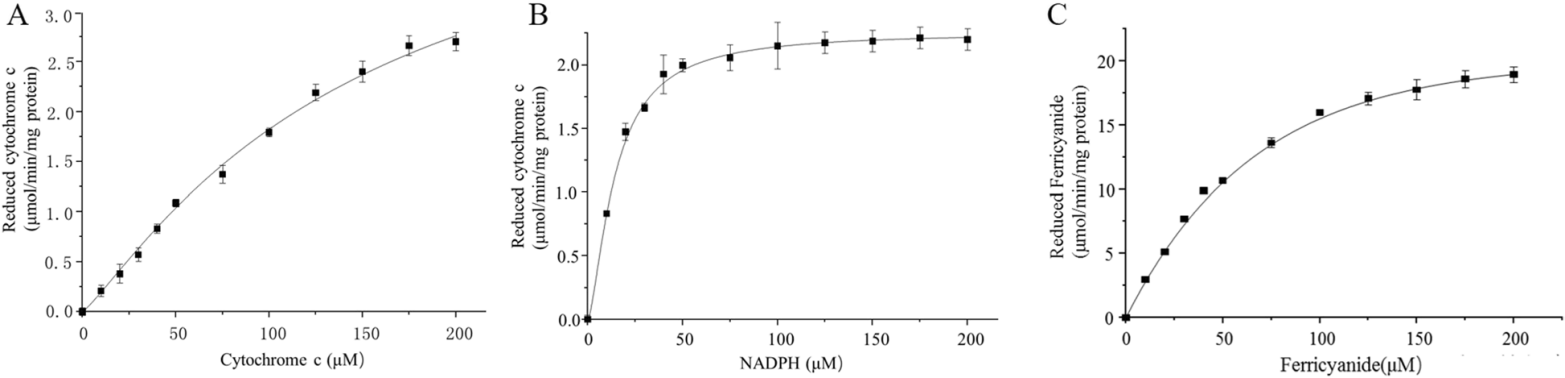
Enzyme kinetic analysis of recombinant proteins MaCPRs. **A** and **B** Kinetic parameters of recombinant protein MaCPR2 on cytochrome *c* and NADPH were calculated by measuring the change in absorbance at 550 nm. **C** Kinetic parameters of recombinant protein MaCPR2 on K_3_Fe(CN)_6_ were calculated by measuring the change in absorbance at 424 nm

The purified recombinant MaCPR proteins were used to conduct enzyme assays. Firstly, the electron transfer activity of the MaCPR protein for cytochrome *c* was determined (Fig. 4A). The MaCPR2-catalyzed reduction of cytochrome *c* gradually increased with increasing cytochrome *c* concentration, and finally reached equilibrium. The results showed that MaCPR2 can transfer electrons to cytochrome *c*, thus leading to the reduction of cytochrome *c* as the reaction substrate of MaCPR2 protein. The activity of MaCPR protein on NADPH was investigated (Fig. 4B). As NADPH concentration in the reaction system was elevated, the amount of cytochrome *c* catalyzed by MaCPR2 also gradually increased, and the reaction curve trend eventually leveled off. This suggests that NADPH can be used as an electron donor for the MaCPR2 protein. Finally, the electron transfer activity of MaCPR protein to K_3_Fe(CN)_6_ was determined (Fig. 4C). The reduced K_3_Fe(CN)_6_ generated by MaCPR2 showed an increase corresponding to the elevation of K_3_Fe(CN)_6_ content in the reaction system, until reaching equilibrium. The results showed that MaCPR2 catalyzed K_3_Fe(CN)_6_ conversion towards the reduced ferricyanide compounds in the situation where K_3_Fe(CN)_6_ served as the substrate. Unfortunately, MaCPR1 did not show any catalytic activity toward cytochrome *c* and K_3_Fe(CN)_6_ under our experimental conditions. Furthermore, the kinetic profile of MaCPR2 in response to NADPH, cytochrome *c*, and K_3_Fe(CN)_6_ follows typical Michaelis Menten curves. The Km values of MaCPR2 for cytochrome *c*, NADPH and K_3_Fe(CN)_6_ were 278, 13.93, and 70.83, respectively (Table 2).

**Table 2:** Steady-state kinetic constants of recombinant protein MaCPR2. All data represent the mean ± SD of three independent replicated trials

		**Vmax (μmol/min/mg)**	**Km (μmol/L)**
Cytochrome *c*	MaCPR2	6.74 ± 0.65	278 ± 40.59
NADPH	MaCPR2	2.43 ± 0.05	13.93 ± 1.54
K_3_Fe(CN)_6_	MaCPR2	26.26 ± 0.14	70.83 ± 4.34

### MaCPR2 exhibit CYP450 monooxygenase activity

To investigate the potential electron transfer function of MaCPR2 for CYP450, we assessed its interaction with MaC3'H hydroxylase. The hydroxylation function of MaC3'H has been previously established in our research on mulberry leaves [28, 29].

**Fig. 5:**
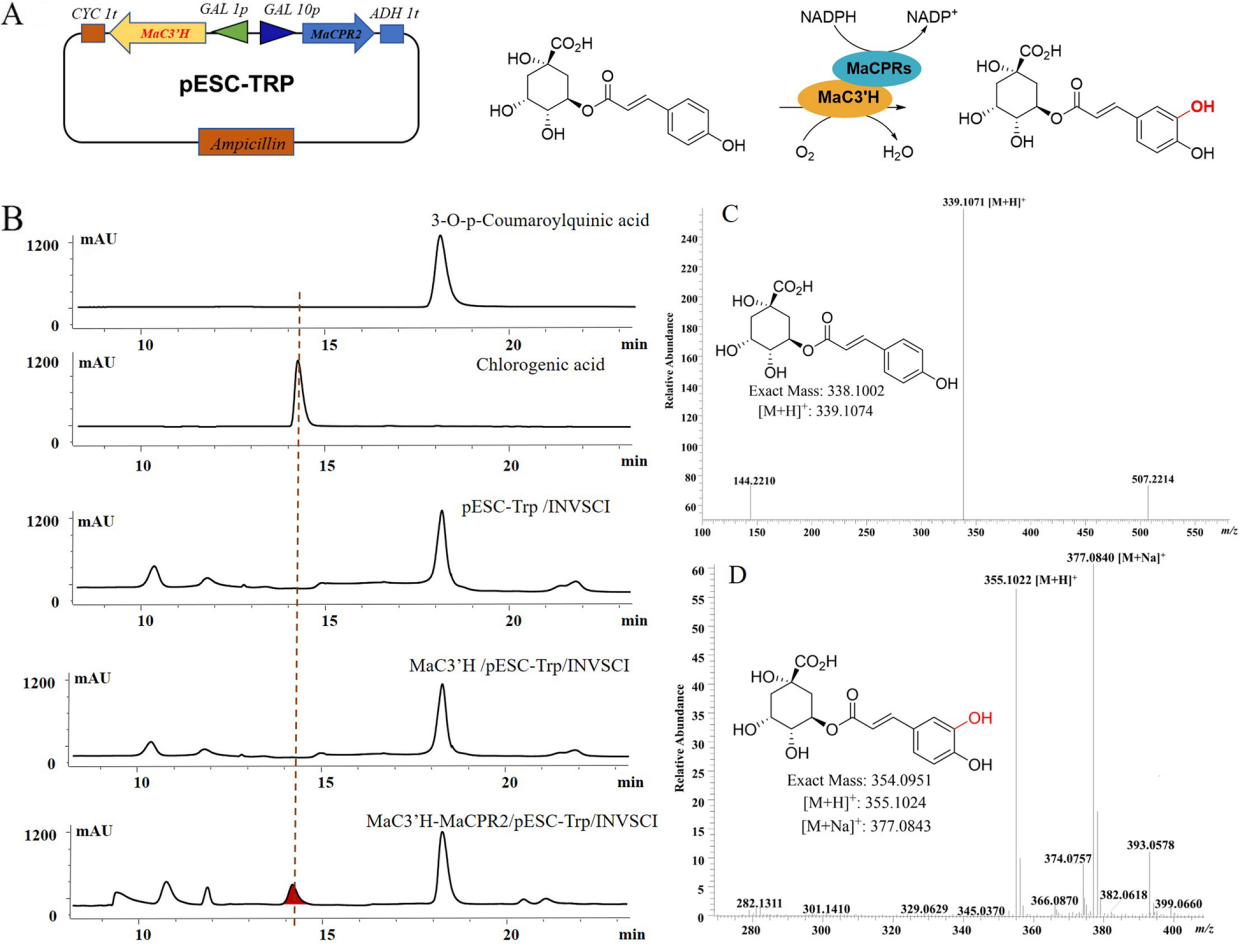
HPLC–MS analysis of MaCPR2 exhibit MaC3'H activity in yeast. **A** Schematic representation of the enzymatic reaction of recombinant protein MaCPR2 on electron transfer to MaC3'H hydroxylase. **B** HPLC analysis of metabolites extracted from transgenic yeast strains. MaC3'H, pESC-Trp, ND. **C** and **D** Mass spectrogram analysis of yeast co-expressed metabolites

The ORFs sequences of *MaCPR2* and *MaC3'H* genes were cloned, ligated with the eukaryotic expression vector pESC-Trp (Fig. 5A), and transformed into yeast NVSCI to construct heterologous co-expression yeast engineering bacterium (Supplementary figure S2). HPLC showed that the expected product chlorogenic acid was detected in MaC3'H-MaCPR2/pESC-Trp/INVSCI engineered bacteria with the same retention time as the reference standard (Fig. 5B). However, no corresponding products were found in yeast extracts of pESC-Trp/INVSCI and MaC3'H/pESC-Trp/INVSCI engineered bacteria (Fig. 5B). The results of MS showed that there was a molecular ion peak *m/z* = 355 in the yeast extract containing MaC3'H-MaCPR2/pESC-Trp/INVSCI in positive ion mode, and a molecular ion peak *m/z* = 353 in negative ion mode (Supplementary figure S9). HRMS analysis of the yeast extract revealed that the exact masses of chlorogenic acid [M + H]^+^ and [M + Na]^+^ were *m/z* 355.1022 and 377.0840, respectively (Fig. 5D), and the exact mass of 3-O-p-coumaroylquinic [M + H]^+^ was *m/z* 339.1071 (Fig. 5C). As reported previously, the product was identified as chlorogenic acid [30]. The above results indicated that MaCPR2 supports the activity of CYP450 monooxygenase as well as transfers electrons to MaC3'H hydroxylase, thus transforming 3-O-p-coumaroylquinic acid into chlorogenic acid.

## Discussion

DNJ belongs to the piperidine class of polyhydroxy alkaloids. Due to its unique structure and significant pharmacological activities, DNJ has been attracting much attention. Although there are two speculations about the biosynthetic pathway of DNJ in mulberry leaves, the mining of CPR functional genes in mulberry leaves is the key to achieving the heterologous synthesis of DNJ, whether it originates from lysine or glucose [12–14]. In the biosynthetic pathway originating from lysine, 2-methylpiperidine is stereoselectively hydroxylated by CYP450 hydroxylase to form DNJ [12]. During this process, cytochrome P450 reductases, act as a redox partner of CYP450 and are major factors in its biological activity [21]. In the biosynthetic pathway originating from glucose, the biochemical processes of functional enzymes, such as reduction or isomerization, also require the participation of electron-donating systems [9, 13]. The main reason is that many isomerase enzymes in the biosynthesis pathway of plant secondary metabolites are also responsible for CYP450 [31, 32]. Therefore, the mining of CPR functional genes in mulberry leaves is of great importance in elucidating the biosynthetic pathway of DNJ.

Yeast and mammals contain only one CPR [33, 34], while plants may contain 1–4 paralogous genes for CPRs [26, 35]. A single CPR gene has been reported to be present in *Coleus blumei*, *Papaver somniferum*, and *Vigna radiata*. Two CPR paralogous genes have been reported to be present in *Capsicum annuum*, *Arabidopsis thaliana*, *Camptotheca acuminata*, *Petroselinum crispum*, and *Siraitia grosvenorii*. Four CPR paralogous genes have been reported to be present in *Andrographis paniculate* [27, 34, 36]. Some of the CPR genes in these different plants can transfer electrons to their corresponding CYP450 hydroxylase, thereby enhancing the catalytic activity of CYP450 hydroxylase. For example, the hydroxylation activity of CYP98A14 was increased seven-fold by co-expressing *Coleus blumei*-CPR and CYP98A14 in yeast [37]. The catalytic activity of geraniol 10-hydroxylase (G10H) was increased 11-fold when geraniol 10-hydroxylase (G10H) was co-expressed with NfCPR2 in insect cells [38]. In this study, two MaCPR genes, MaCPR1 and MaCPR2, were screened from the transcriptome data of mulberry leaves. The sequence analysis showed that they had complete ORFs with MaCPR1 and MaCPR2 lengths of 2064 bp and 2148 bp, respectively. Phylogenetic tree analysis revealed that MaCPR1 and MaCPR2 were found to cluster into two distinct branches, MaCPR1 belongs to class I, while MaCPR2 belongs to class II. Class II-induced expression of CPR participates in induced specialized metabolism, plays a role in an adaptive mechanism or defense response (such as trauma, pathogen infection, etc.), and transfers electrons for CYP450s involved in specialized metabolism to induce biosynthesis of specialized metabolites [39, 40]. Our previous studies clarified the molecular mechanism of low-temperature stress on the changes of chemical components in mulberry leaves, also revealing the material basis for the formation of high-quality mulberry leaves after frost [41]. In this study, our findings reveal that, MaCPR2 possesses an electron transfer function, which is crucial for hydroxylases within the biosynthetic pathway of secondary metabolites in mulberry leaves. It provides additional substantiation that the intricate secondary metabolites in mulberry leaves serve as elaborate defense mechanisms, evolving to adapt and ensure survival amidst biotic, or abiotic stress, during the evolutionary process [42]. The biological functions of this gene in response to various stresses in mulberry leaves will be further explored.

Cytochrome P450 reductases have a transmembrane helical structural domain at its N-terminus that is anchored to the endoplasmic reticulum [43, 44]. Its recombinant protein will localize to the bacterial membrane system in CPR heterologous expression, which in turn affects the solubility of the recombinant protein, and the membrane-anchored region is not required for recombinant protein activity [22, 23]. Therefore, to improve the solubility of the recombinant proteins, the original transmembrane regions in the amino acid sequences of the MaCPRs were deleted when the recombinant MaCPRs were expressed in *E. coli* DH5α to be present in a soluble state in the supernatant of the cell-breaking solution. In vitro enzyme activity studies revealed that MaCPR2 could use NADPH as an electron donor and react with cytochrome *c*/ K_3_Fe(CN)_6_ as the reaction substrate to generate reduced cytochrome *c* or reduced ferricyanide. However, no similar function was found for MaCPR1 under this experimental condition.

The research on the biosynthetic pathways of plant natural products usually utilizes the substrate-feeding method of engineered bacteria, which commonly used engineered bacteria are *E. coli* and yeast [45, 46]. It has been demonstrated that, lacking the endoplasmic reticulum, *E. coli* becomes unsuitable for CYP450 expression. Conversely, yeast is a commonly employed platform for CYP450 expression. We used transgenic yeast INVSCI containing the eukaryotic expression vector pESC-Trp/MaC3'H-MaCPR2 as chassis cells. Yeast feeding experiments using 3-O-p-coumaroylquinic acid as substrate. It was found that MaCPR2 could transfer electrons to MaC3'H hydroxylase, which would perform a mono-oxygenation reaction of 3-O-p-coumaroylquinic acid to produce chlorogenic acid, thus exerting the hydroxylation function of hydroxylase. However, in the present study, the electron transfer function of the MaCPR2 protein was verified only using MaC3'H hydroxylase from mulberry leaves, which is involved in the chlorogenic acid biosynthesis pathway. Subsequently, MaCPR2 was co-expressed with CYP450s of the DNJ biosynthesis pathway in *S. cerevisiae* to analyze the effects of MaCPRs on the catalytic functions of different CYP450s.

## Conclusion

In summary, we cloned and characterized two MaCPR genes from the transcriptome profile of mulberry leaves, in which the recombinant protein MaCPR2 was able to reduce cytochrome *c* and ferricyanide using NADPH as an electron donor. We co-expressed MaCPR2 and MaC3'H hydroxylase in yeast, to illustrate that MaCPR2 has an electron transfer function for MaC3'H hydroxylase, enabling MaC3'H hydroxylase to catalyze the generation of chlorogenic acid from 3-O-p-coumaroylquinic acid. The above findings reveal that MaCPR2 might be involve in the biosynthesis of primary and secondary metabolites in mulberry leaves. Currently, we are analyzing the related CYP450 in the DNJ biosynthesis pathway as well as validating the in vivo biological functions of the identified MaCPRs in mulberry leaves, to further elucidate the mechanism of DNJ biosynthesis in mulberry leaves.

### Supplementary Information

**Supplementary Material 1.**

## Data Availability

Nucleotide sequences reported in this study were submitted to the National Center for Biotechnology Information (NCBI) with accession numbers: *MaC3'H* (MK738016), *MaCPR1* (OR995401), *MaCPR2* (OR995402). The datasets generated during the current study are available in the NCBI database under SRA accession repository: SRP127713 (https://www.ncbi.nlm.nih.gov/sra/?term=SRP127713).

## Abbreviations

DNJ: 1-Deoxynojirimycin
CPRs: NADPH-cytochrome P450 reductases
MaLDC: Lysine decarboxylase
MaCAO: Copper amine oxidase
MaSDRs: Reductases
MaMTs: Methyltransferases
CYP450s: Cytochrome P450 enzymes
NCBI: National Center for Biotechnology Information
IPTG: Isopropyl-*β*-D-thiogalactopyranoside
PMSF: Phenylmethylsulfonyl fluoride
MaC3'H: Ester 3'-hydroxylase
HPLC: High performance liquid chromatography
HRMS: High resolution mass spectrometry
