# Supplementary material for: Mining and functional characterization of NADPH-cytochrome P450 reductases of the DNJ biosynthetic pathway in mulberry leaves
Source: BMC Plant Biol. 2024 Feb 23;24:133. doi: 10.1186/s12870-024-04815-0 (PMC10885410; doi:10.1186/s12870-024-04815-0)
Supplement: Supplementary file 1 — Supplementary Material 1. [file 12870_2024_4815_MOESM1_ESM.docx]

**Supplementary information**

**Mining and functional characterization of NADPH-cytochrome P450 reductases of the DNJ biosynthetic pathway in mulberry leaves**

Yangzhen Liao ^1^, Wenmin Du ^2^, Jingqiong Wan ^2^, Jiahe Fan ^1^, Jilan Pi ^2^, Min Wu ^2^, Yuan Wei ^2^, and Zhen Ouyang ^1, 2,^ *

^1^ School of Food and Biological Engineering, Jiangsu University, Zhenjiang 212013, PR, China

^2^ School of Pharmacy, Jiangsu University, Zhenjiang 212013, PR, China

*Correspondence author: Dr. Zhen Ouyang. Tel: +86-0511-88791564. E-mail: zhenouyang@ujs.edu.cn.

**Supplementary figure S1. Agarose gel electrophoresis results of PCR products of truncated *MaCPR1* (A) and *MaCPR2* (B) genes.** Lane M: DNA ladder (0.1-5 kb); Lane 1: PCR product of *MaCPR1*; Lane 2: PCR product of *MaCPR2.*

**Supplementary figure S2. Agarose gel electrophoresis results of PCR products of full-length *MaCPR2* and full-length *MaC3'H* genes.** Lane M: DNA ladder (0.1-5 kb); Lane 1-3: PCR product of *MaC3'H*; Lane 4-6: PCR product of *MaCPR2.*

**Supplementary figure S3. Signal peptide prediction of MaCPR1 (A) and MaCPR2 (B) proteins.**

**Supplementary figure S4. Prediction of transmembrane domains of MaCPR1 (A) and MaCPR2 (B).**

**Supplementary figure S5. Secondary structure prediction of MaCPR1 (A) and MaCPR2(B) proteins.**

**Supplementary figure S6. Tertiary structure prediction of MaCPR1 (A) and MaCPR2 (B) proteins.**

**Supplementary figure S7. SDS-PAGE diagram of protein expressed by *E. coli* BL21 (DE3) containing recombinant plasmid MaCPRs/pET-32a(+). (A)** M: Protein molecular weight standard (10-170 kDa); 1. Uninduced protein of *E. coli* BL21 (DE3) containing MaCPR1/pET-32a(+); 2. Protein induced by *E. coli* BL21 (DE3) containing MaCPR1/pET-32a(+); **(B)** M: Protein molecular weight standard (10-170 kDa); 1. Uninduced protein of *E. coli* BL21 (DE3) containing MaCPR2/pET-32a(+); 2. Protein induced by *E. coli* BL21 (DE3) containing MaCPR2/pET-32a(+).

**Supplementary figure S8. BCA method protein determination standard curve.**

**Supplementary figure S9. Mass spectrometry analysis of yeast extract from MaC3'H-MaCPR2/pESC-Trp/INVSCI engineering bacteria** (A: positive ion mode, B: negative ion mode).

**Supplementary figure S10. Full uncropped Gels images. (A) Fig 3A full uncropped gel image. (B) Fig 3B full uncropped gel image.** Lane M: Protein molecular markers (10-170 kDa); Lane 1, 10: Supernatant after lysis of *E. coli* BL21 (DE3); Lane 2, 11: Flow through liquid; Lane 3-9, 12-18: Imidazole eluent at different concentrations.

**Supplementary table S1. Nucleotide sequences and protein sequence of MaCPR1.**

**Supplementary table S2. Nucleotide sequences and protein sequence of MaCPR2.**

**Supplementary table S3. Nucleotide sequences and protein sequence of MaC3'H.**

**Supplementary table S4. PCR reaction systems of *MaCPR1* and *MaCPR2* genes.**

**Supplementary table S5. PCR reaction conditions of *MaCPR1* genes.**

**Supplementary table S6. PCR reaction conditions of *MaCPR2* genes.**

**
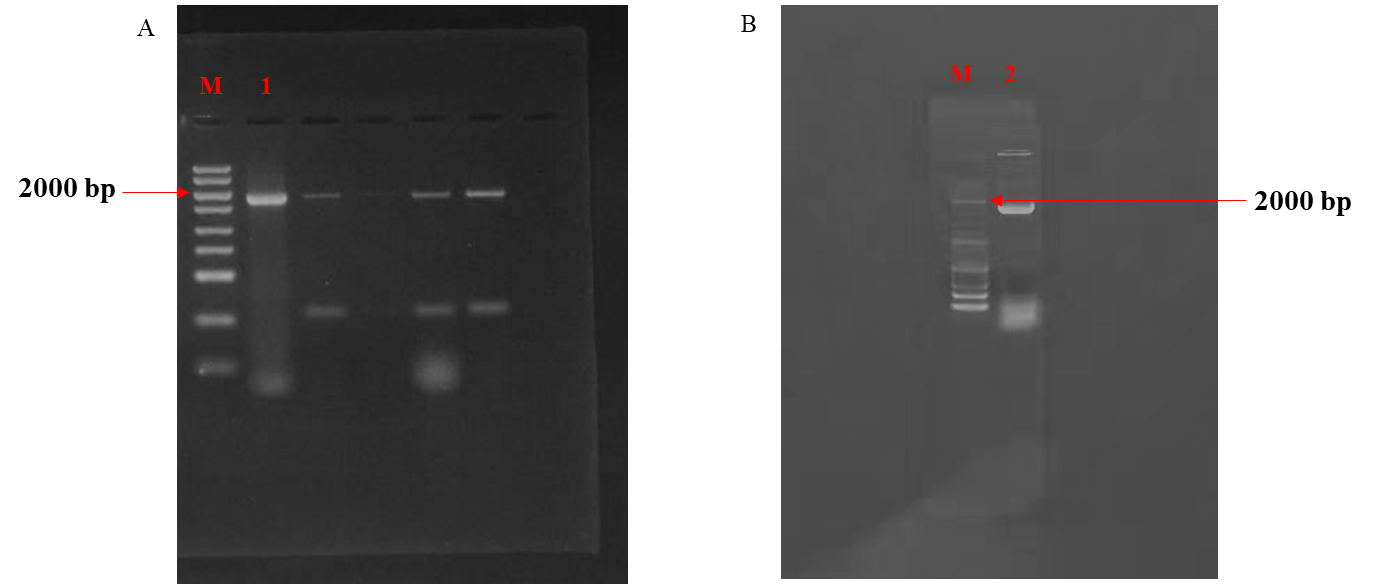
**

**Supplementary figure S1. Agarose gel electrophoresis results of PCR products of truncated *MaCPR1* (A) and *MaCPR2* (B) genes.** Lane M: DNA ladder (0.1-5 kb); Lane 1: PCR product of *MaCPR1*; Lane 2: PCR product of *MaCPR2.*

**
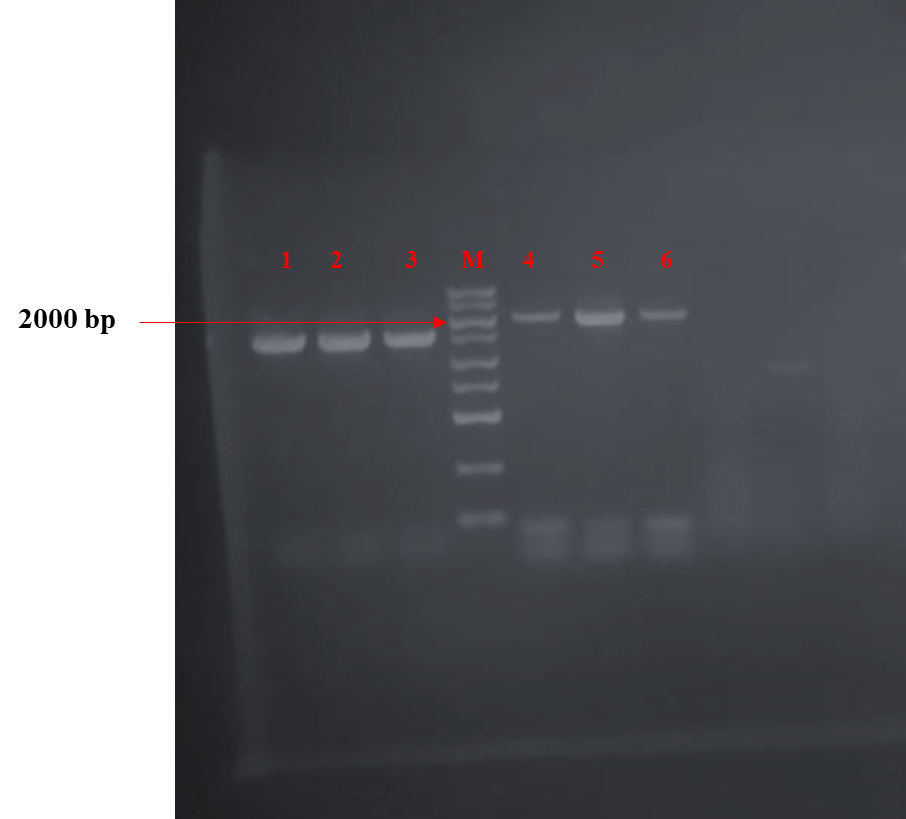
**

**Supplementary figure S2. Agarose gel electrophoresis results of PCR products of full-length *MaCPR2* and full-length *MaC3'H* genes.** Lane M: DNA ladder (0.1-5 kb); Lane 1-3: PCR product of *MaC3'H*; Lane 4-6: PCR product of *MaCPR2.*


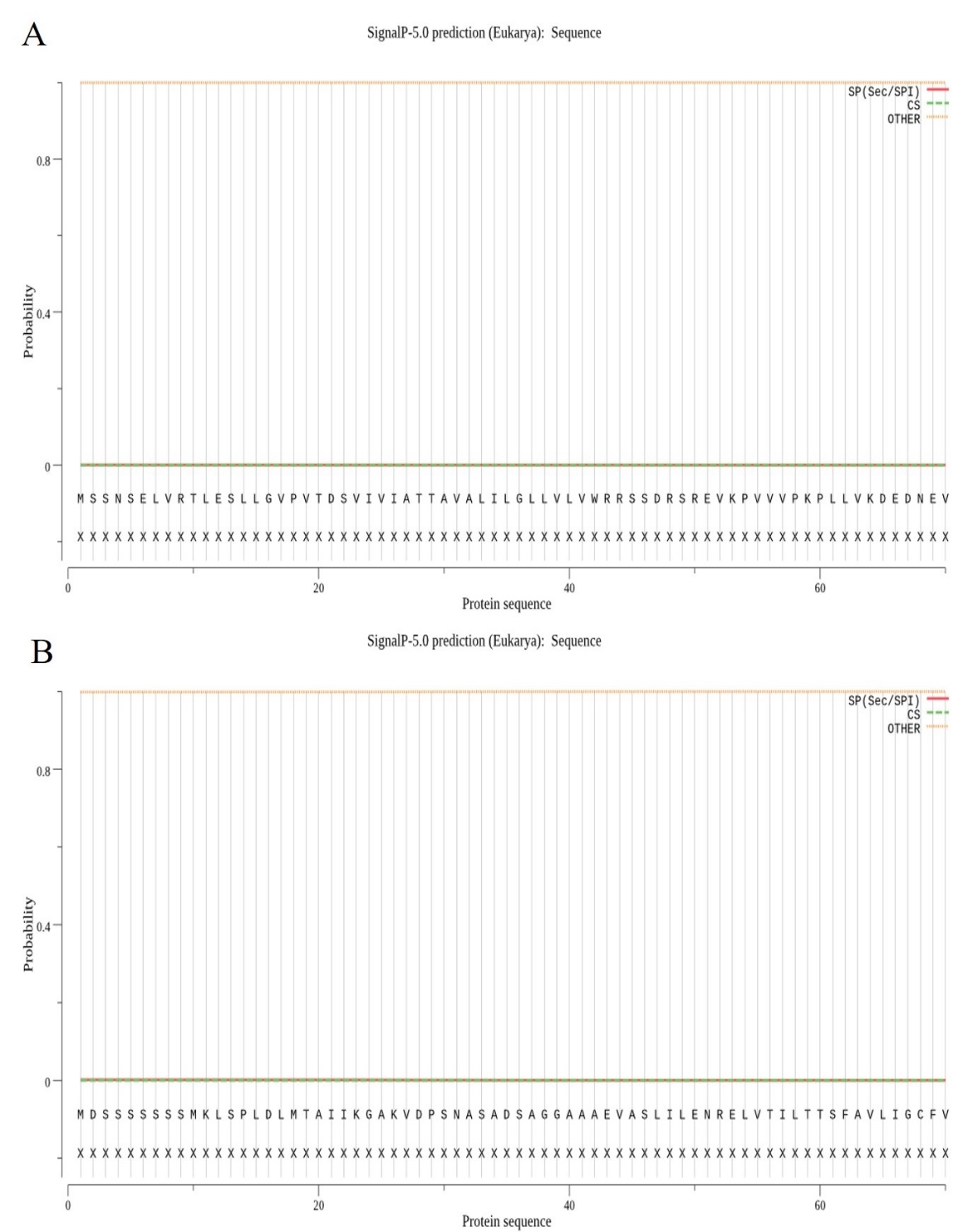


**Supplementary figure S3. Signal peptide prediction of MaCPR1 (A) and MaCPR2 (B) proteins.**


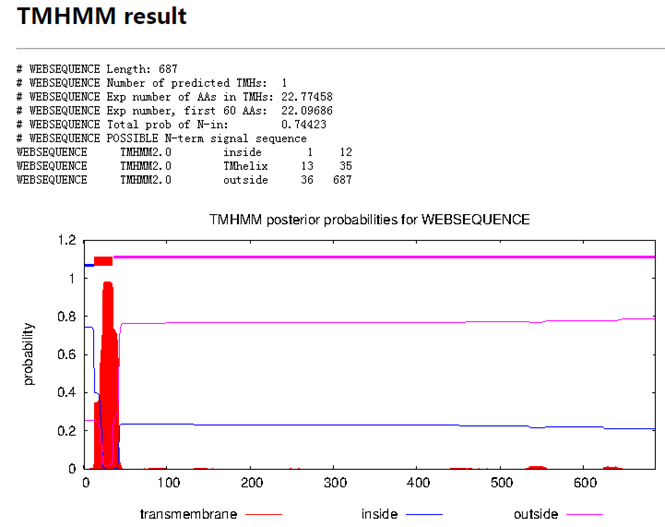


A

B


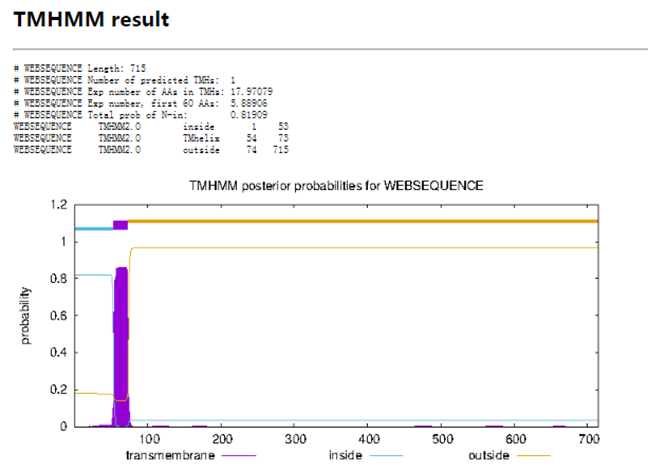


**Supplementary figure S4. Prediction of transmembrane domains of MaCPR1 (A) and MaCPR2 (B).**


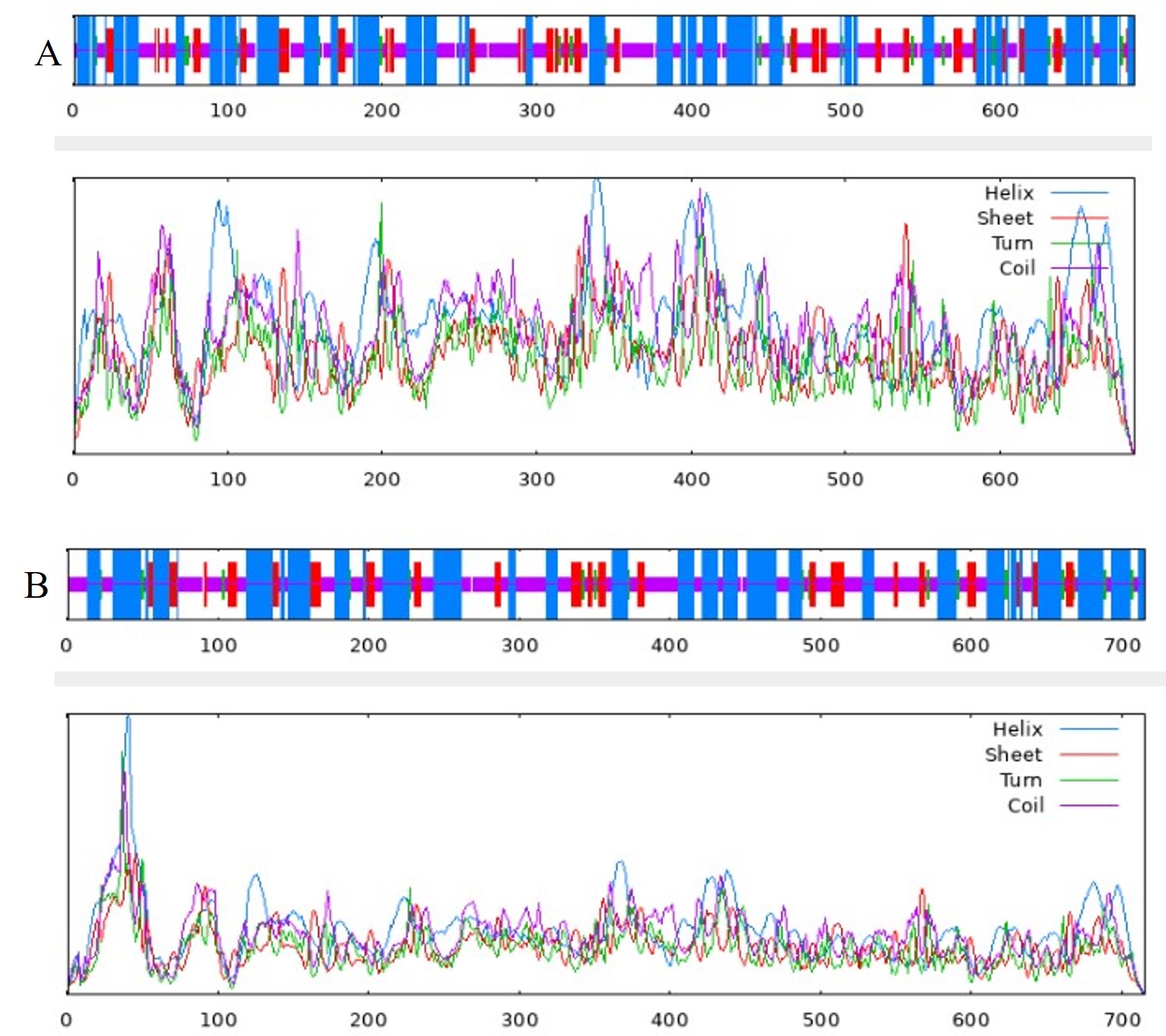


**Supplementary figure S5. Secondary structure prediction of MaCPR1 (A) and MaCPR2(B) proteins.**


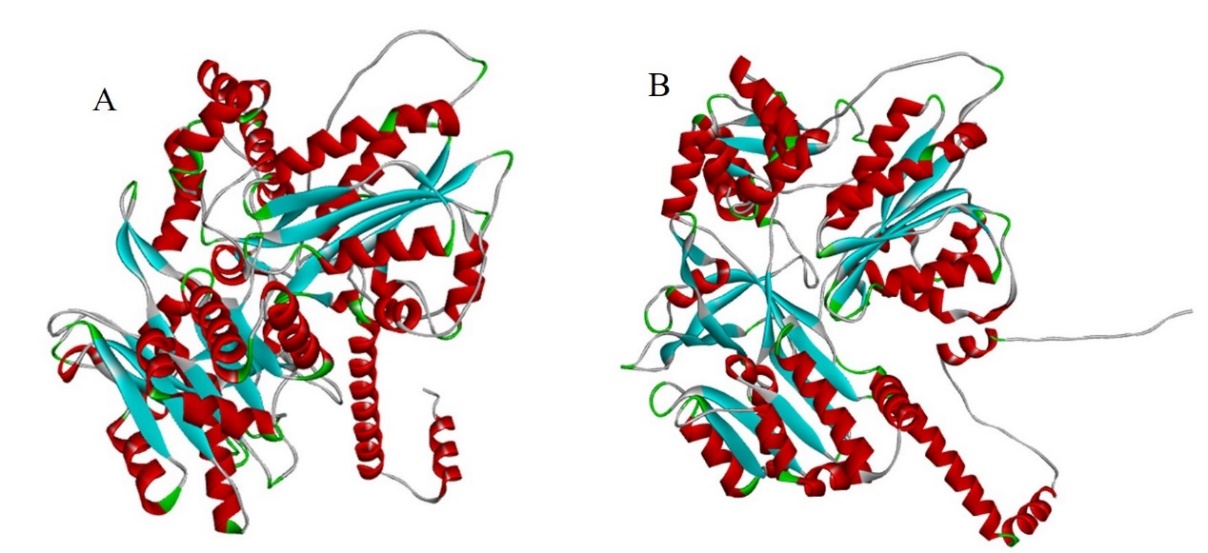


**Supplementary figure S6. Tertiary structure prediction of MaCPR1 (A) and MaCPR2 (B) proteins.**


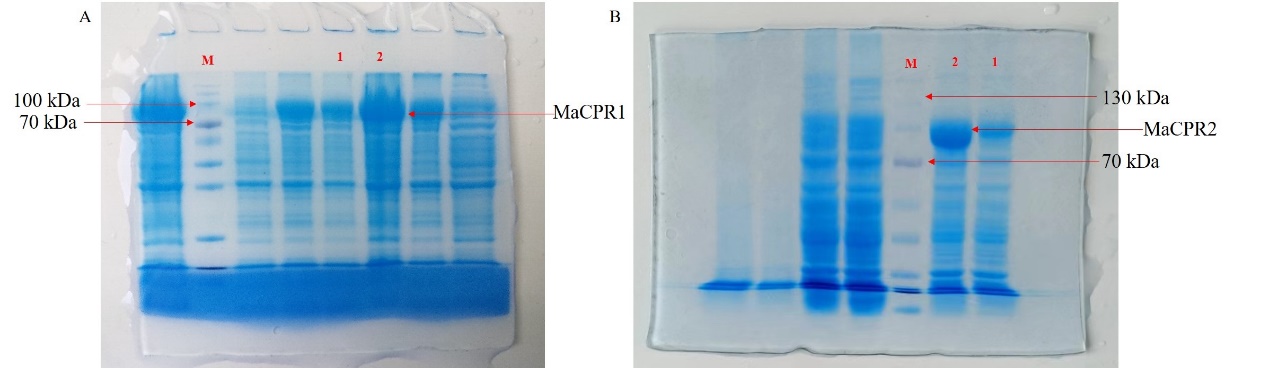


**Supplementary figure S7. SDS-PAGE diagram of protein expressed by *E. coli* BL21 (DE3) containing recombinant plasmid MaCPRs/pET-32a(+). (A)** M: Protein molecular weight standard (10-170 kDa); 1. Uninduced protein of *E. coli* BL21 (DE3) containing MaCPR1/pET-32a(+); 2. Protein induced by *E. coli* BL21 (DE3) containing MaCPR1/pET-32a(+); **(B)** M: Protein molecular weight standard (10-170 kDa); 1. Uninduced protein of *E. coli* BL21 (DE3) containing MaCPR2/pET-32a(+); 2. Protein induced by *E. coli* BL21 (DE3) containing MaCPR2/pET-32a(+).


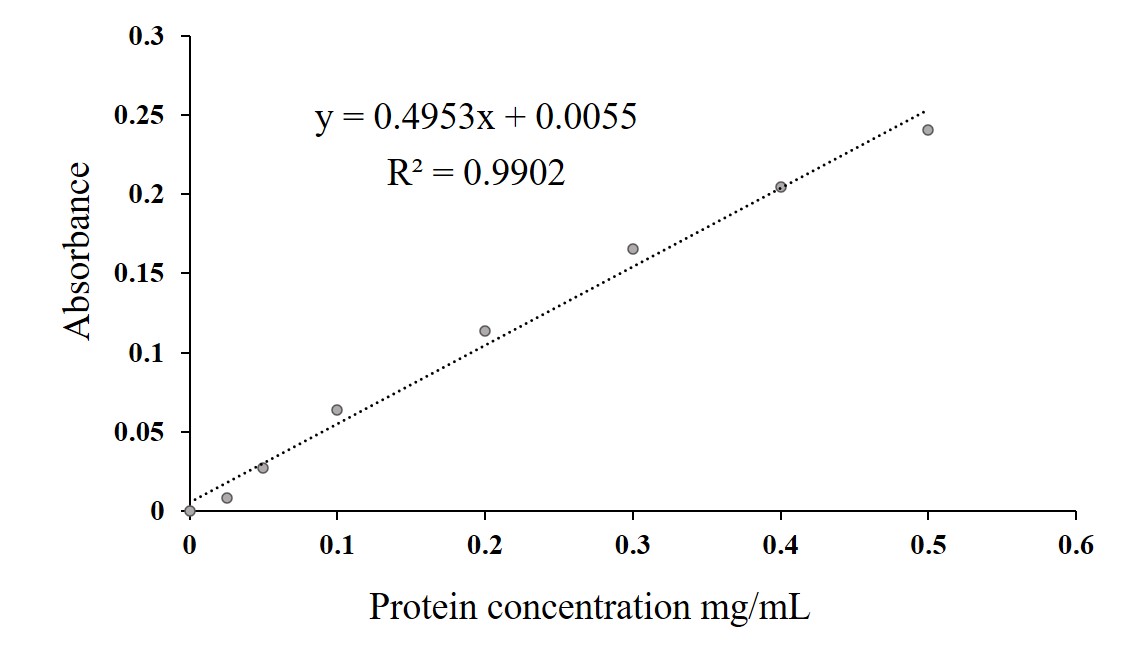


**Supplementary figure S8. BCA method protein determination standard curve.**


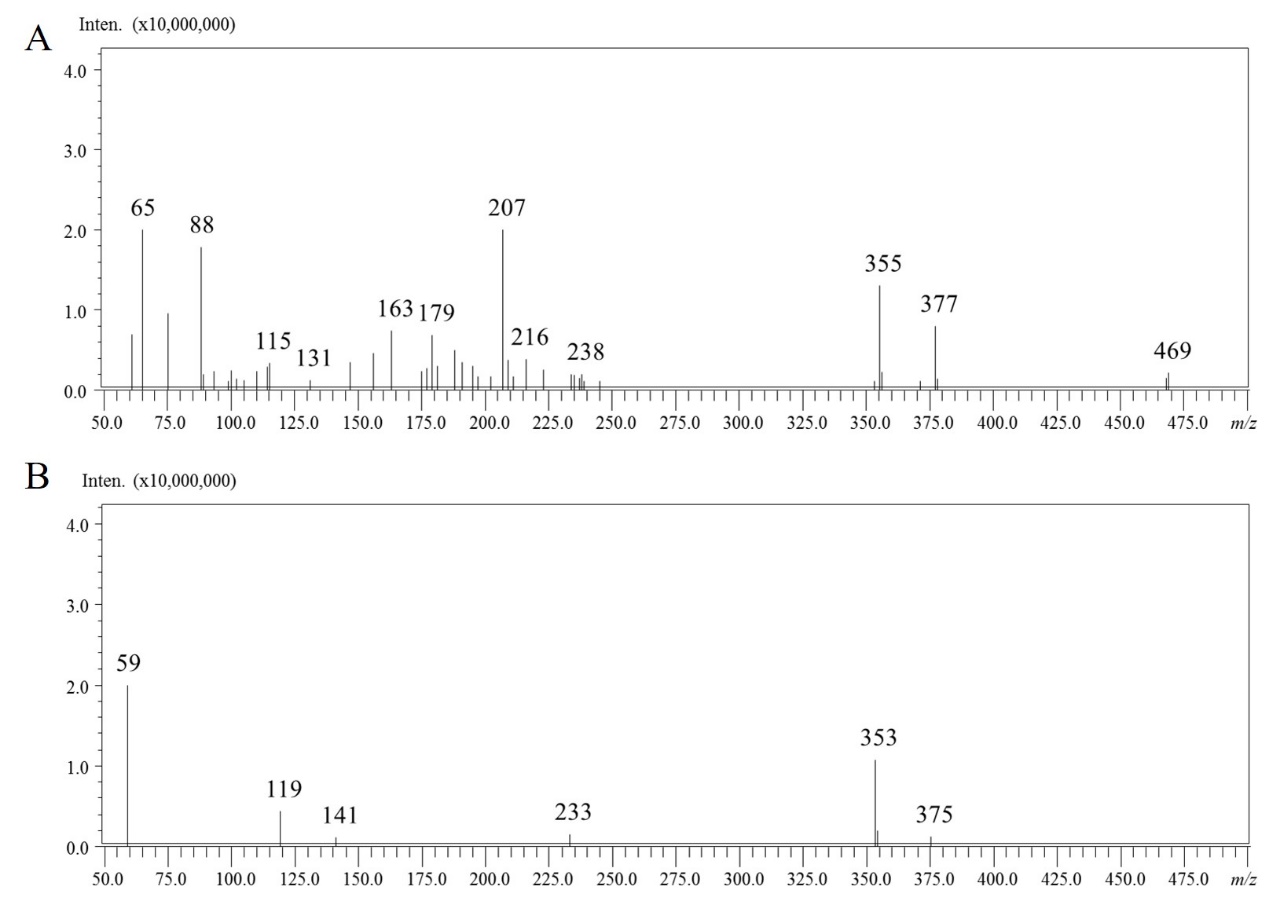


**Supplementary figure S9. Mass spectrometry analysis of yeast extract from MaC3'H/pESC-Trp/INVSCI engineering bacteria** (A: positive ion mode, B: negative ion mode).


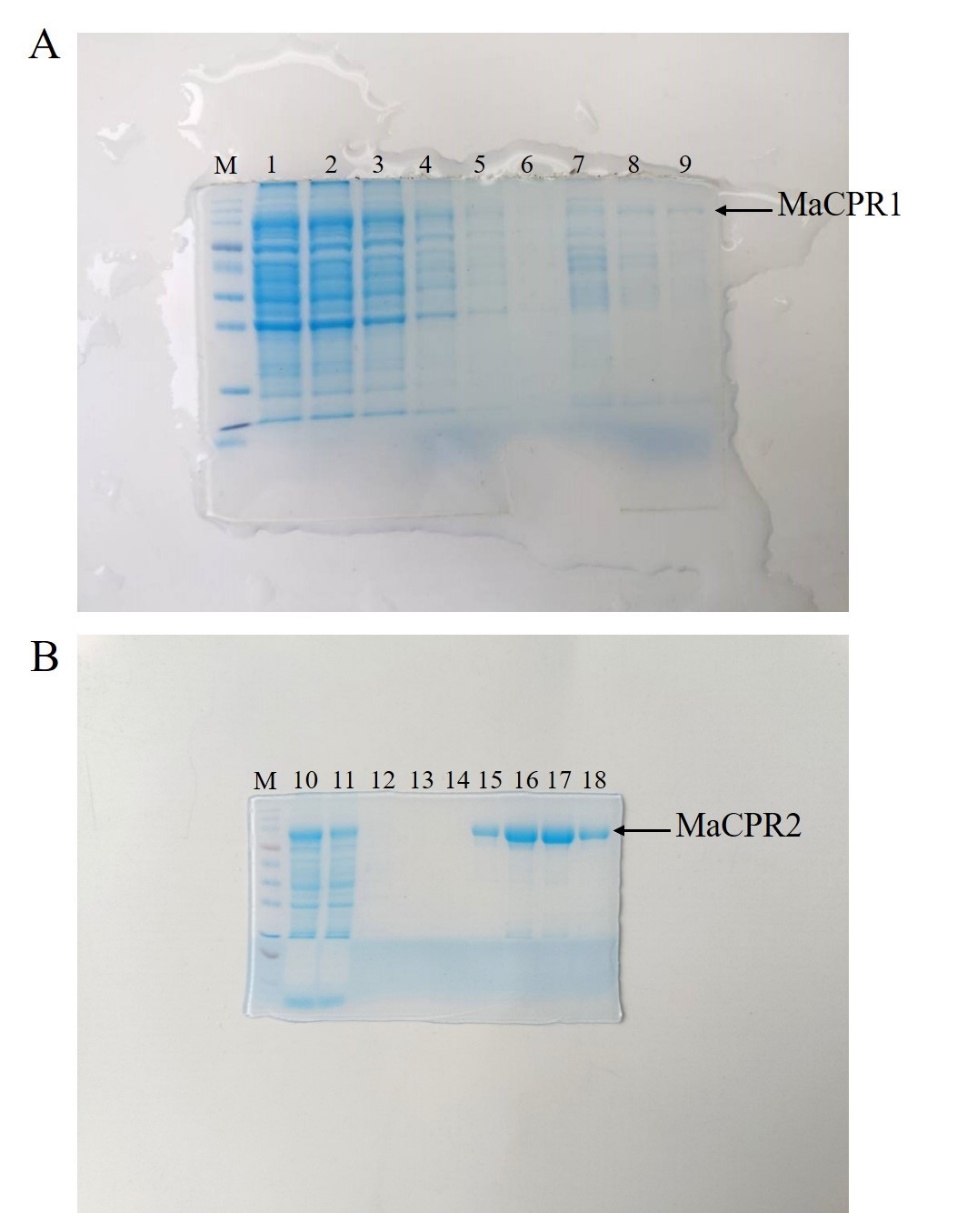


**Supplementary figure S10. SDS-PAGE analysises of purified recombinant protein MaCPR1 and MaCPR2. (A) Fig 3A full uncropped gel image. (B) Fig 3B full uncropped gel image.** Lane M: Protein molecular markers (10-170 kDa); Lane 1, 10: Supernatant after lysis of *E. coli* BL21 (DE3); Lane 2, 11: Flow through liquid; Lane 3-9, 12-18: Imidazole eluent at different concentrations.

**Supplementary table S1. Nucleotide sequences and protein sequence of MaCPR1.**

| **Nucleotide sequences** | >Seq1 [*Morus alba* L.] *MaCPR1*  ATGAGTTCGAATTCGGAGCTGGTGCGAACTCTGGAGTCTTTGCTTGGGGTTCCGGTGACGGACTCCGTGATTGTGATCGCCACGACCGCCGTGGCGCTGATTCTCGGACTTCTGGTGTTGGTTTGGAGGAGATCGTCGGATCGGAGCAGAGAGGTGAAGCCGGTGGTGGTGCCGAAGCCACTACTGGTCAAGGACGAAGACAACGAGGTTGAGCTCGCCTCTGGCAAGACCAAGGTCACTGTTTTTTATGGAACTCAGACTGGTACGGCCGAGGGATTTGCTAAGGCTTTAGCAGAAGAGATCAGGGCAAGATATGAAAAAGCAGTTGTCAAAGTTGTTGACATGGATGATTATGCTGTGGATGATGATCAATATGAGGAAAAACTTAAGAAAGAGACTCTGGCGTTTTTCATGGTGGCCACTTATGGAGATGGAGAACCTACTGATAATGCAGCAAGATTTTACAAATGGTTTACTGAGGGAGACAATAAGGGTAGCTGGCTTCAACAACTCAGATATGGCGTTTTTGGTTTGGGTAACCGACAATATGAACATTTCAATAAGATTGGGAAGGTGATTGATGAGCAACTGAGTGAACAAGGTGCAAAGCGTCTCGTTCCACTTGGCCTAGGTGATGATGATCAGTGCATTGAGGATGATTTTTCTGCTTGGAGAGAATTGTTGTGGCCTGAGTTGGATCAAATACTTCGAGATGAAGATGATACAACTACTGTATCTACTCCATATACAGCTGCAGTCCCTGAGTATTGCGTTGTTATTCATGATCCTAGTGTTGCCAATTTTGAGGATAATTACTTAAGTATGCCGAATGGAAACGCTTCTTTTGATATTCACCATCCTTGCAGAGTAAATGTCGCTGTCCAAAAAGAACTTCACAAGCCCGAGTCTGACAGATCCTGCATACATTTGGAATTTGATATATCTGGAACTGGTATAAAATATGAAACAGGAGACCATGTGGGTGTTTATGCTGAGAATTGCGATGAAACCATTGAAGAGGCTGCAAAATTGTTGGGTCAACCTTTAGATCTTCTATTCTCTATCCACACTGACAATGAGGATGGCTCTCCCCTTGGGAATTCATTGCCTCCACCTTTCCCTGGTCCGTGCACACTGCGCAGTGCATTGGCACGTTATACTGATCTGTTAAACCCACCTCGAAAAGCTGCTCTAATTGCTTTGGCTGCACATGCTGTTGAACCGAGTGAAGCAGAAAAACTTAAGTTCTTATCATCACCTCAGGGGAAGGATGAGTACTCAAAATGGGTTGTTGGAAGTCAAAGAAGTCTTCTTGAGATTATGGCTGAGTTCCCGTCAGCAAAGCCTCCACTTGGTGTGTTTTTTGCTGCAGTAGCCCCTCACTTGCAGCCTCGTTACTACTCAATCTCATCTTCACCAAGGTTTGCCCCCCATCGTGTTCATGTTACATGCGCTTTGGTATATGGTCCAAGTCCTACTGGAAGAATTCACAAAGGAGTGTGTTCAACCTGGATGAAGAATGCAGTGCCTCTTGAGAAGAGCCAAAATCCTAGCTGGGCTCCTATTTTTATTAGGCAATCTAATTTCAAGTTACCAGCTGATCATTCAATCCCAGTTATCATGGTGGGACCTGGAACTGGCTTAGCACCTTTCAGAGGATTTTTACAGGAAAGAATGGCCCTCAAAGAGGAGGGTGCTCAACTTGGTCCTGCACTTCTATTCTTTGGATGTAGAAATCGCCGAATGGATTTTATTTATGAGGATGAGCTAAACAACTTTGTTGAACAAGGTGCAATGTCAGAATTGATTGTTGCGTTCTCACGAGAGGGGCCGGAAAAAGAGTATGTTCAACATAAGATGATGGATAAAGCATCTTGTATATGGAGCCTGATATCACAAGGGGGATACTTCTACGTATGTGGTGATGCCAAAGGCATGGCTAGAGATGTTCATCGGACTTTGCATACCATAGTCCAACAACAGGAAAACGTGGACTCATCAAAGGCAGAATCTATAGTGAAGAAACTCCAGATGGACGGACGATATCTGAGAGATGTCTGGTGA |
| --- | --- |
| **Protein Sequence** | >Seq1 [*Morus alba* L.] MaCPR1  MSSNSELVRTLESLLGVPVTDSVIVIATTAVALILGLLVLVWRRSSDRSREVKPVVVPKPLLVKDEDNEVELASGKTKVTVFYGTQTGTAEGFAKALAEEIRARYEKAVVKVVDMDDYAVDDDQYEEKLKKETLAFFMVATYGDGEPTDNAARFYKWFTEGDNKGSWLQQLRYGVFGLGNRQYEHFNKIGKVIDEQLSEQGAKRLVPLGLGDDDQCIEDDFSAWRELLWPELDQILRDEDDTTTVSTPYTAAVPEYCVVIHDPSVANFEDNYLSMPNGNASFDIHHPCRVNVAVQKELHKPESDRSCIHLEFDISGTGIKYETGDHVGVYAENCDETIEEAAKLLGQPLDLLFSIHTDNEDGSPLGNSLPPPFPGPCTLRSALARYTDLLNPPRKAALIALAAHAVEPSEAEKLKFLSSPQGKDEYSKWVVGSQRSLLEIMAEFPSAKPPLGVFFAAVAPHLQPRYYSISSSPRFAPHRVHVTCALVYGPSPTGRIHKGVCSTWMKNAVPLEKSQNPSWAPIFIRQSNFKLPADHSIPVIMVGPGTGLAPFRGFLQERMALKEEGAQLGPALLFFGCRNRRMDFIYEDELNNFVEQGAMSELIVAFSREGPEKEYVQHKMMDKASCIWSLISQGGYFYVCGDAKGMARDVHRTLHTIVQQQENVDSSKAESIVKKLQMDGRYLRDVW |

**Supplementary table S2. Nucleotide sequences and protein sequence of MaCPR2.**

| **Nucleotide sequences** | >Seq1 [*Morus alba* L.] *MaCPR2*  ATGGACTCCTCGTCGTCGTCGAGCTCGATGAAGCTCTCGCCGTTGGATCTGATGACCGCGATCATCAAAGGCGCGAAAGTGGACCCCTCCAACGCCTCCGCCGACTCTGCCGGCGGTGCCGCCGCCGAGGTCGCCTCGCTCATCCTCGAGAATCGCGAGCTCGTCACGATCCTCACGACGTCGTTCGCCGTCCTGATCGGATGCTTCGTCGTCCTCATGTGGCGGCGATCCGGCTCGCAGAAGCCGAAGGCGGTGGAGCTTCTCAAGCCTTTGGTCGTCAAGGTGCCGGAGATTGAGGCCGACGACGGCAAGAAGAAGGTCACGATCTTCTTCGGGACGCAGACCGGAACCGCCGAGGGCTTTGCCAAGGCGTTGGCTGACGAGGCAAAAGCTCGCTACGAAAAGGCCGTTTTTAAAGTTGTTGATTTGGATGATTATGCGGCCGATGACGATGAATACGAGGAAAAGTTGAAGAAAGAGAGTTTGGCGTTCTTCTTCTTGGCTACATACGGTGATGGTGAGCCAACTGATAATGCTGCAAGGTTTTACAAATGGTTCACAGAGGGTAAAGAGAGAGGGGAATGGCTTCAGAATCCTCAGTACGGAGTGTTTGGTCTCGGTAACAGGCAGTACGAGCATTTCAATAAGGTAGCCAAAGAAGTCGACGATAACCTTACTGAACAGGGTGCGAAGCGCCTTGTGCCTGTGGGTCTTGGAGACGATGATCAATGTATCGAGGATGACTTCACTGCATGGAGAGAATTGGTATGGCCAGAGTTGGATCAGTTGCTTAGAGATGAGGATGATACAACTACTGTTTCTACCCCTTACACTGCTGCTGTATTAGAATACCGTGTTGAATTCCATGACCCTGTTGATGCATCACTAGAGAAAAAGAGCTGGGCTAATGCAAATGGTCATGCTGTCATTGATGCTCAGCACCCATGCAGGGCTAATGTGGCTGTTAGAAGGGAGCTTCATACTCCTGAATCTGATCGTTCCTGTACGCATCCGGAGTTTGACATTGCTAGCACTGGACTTTCTTACGAAACAGGGGACCACGTTGGTGTTTACTGTGAGAATCTTACTGAAGTTGTAGAAGAGGCTCTCAATTTATTGGGCTTGTCTCCTGAAACATACTTTTCCATCCATACTGATAAAGAGGATGGCACACCAATTAGTGGAAGTTCTTTGCCGCCACCTTTCCCGCCCTGCACTCTAAGAACAGCTCTTGCTCAGTATGCAGATCTTTTAAGTTTTCCCAAAAAGTCATCGTTGCTCGCTCTGGCAGCTCATGCTTCTAATCCAGCTGAAGCTGATAGATTAAGACATCTTGCATCACCTGCCGGAAAGGATGAATATGCACAATGGGTGGTTGCAAGTCAGAGAAGCCTTATTGAGGTCATGGCCGAATTTCCTTCAGCCAAGCCCCCACTTGGTGTGTTCTTTGCAGCAGTGGCCCCTCGCTTGCAGCCTAGATACTATTCGATCTCATCATCCCCAAGGATGGCTCCTTCTAGAATTCACGTTACTTGTGCATTAGTTTATGAGAAGACACCGACTGGACGAATTCACAAAGGAGTGTGTTCTACTTGGATGAAGAATGCCGTGCCTGCAGAGAAAAGTGATGATTGTAGTTGGGCACCTACTTTTGTTAGACAGTCGAACTTCAAACTCCCTGCTGATACTAAAGTACCAATAATCATGATTGGCCCTGGTACTGGGTTGGCCCCATTCAGAGGTTTCCTGCAGGAAAGATTAGTTCTAAAAGAATCTGGAGCAGATCTAGGACCATCGATATTGTTCTTCGGATGCAGAAATCGTAGAATGGATTACATTTATGAAGAAGAACTGGCCAACTTTGTAGAAACCGGTGCACTTTCCGAGCTGGTGGTTGCTTTCTCACGTGAGGGACCTACCAAGGAATATGTGCAGCATAAAATGATGCAGAAGGCTGCCGACATATGGGACATGATATCTCAAGGAGCTTACATTTATGTCTGTGGTGATGCCAAGGGCATGGCTAAGGATGTCCACCGAACTCTTCACACTATTGCGCAAGAGCAGGGATCCCTAGACAGCTCCAAGGCTGAGAGCATGGTGAAAAATCTGCAAATGAATGGCAGGTATCTACGTGATGTGTGGTGA |
| --- | --- |
| **Protein Sequence** | >Seq1 [*Morus alba* L.] MaCPR2  MDSSSSSSSMKLSPLDLMTAIIKGAKVDPSNASADSAGGAAAEVASLILENRELVTILTTSFAVLIGCFVVLMWRRSGSQKPKAVELLKPLVVKVPEIEADDGKKKVTIFFGTQTGTAEGFAKALADEAKARYEKAVFKVVDLDDYAADDDEYEEKLKKESLAFFFLATYGDGEPTDNAARFYKWFTEGKERGEWLQNPQYGVFGLGNRQYEHFNKVAKEVDDNLTEQGAKRLVPVGLGDDDQCIEDDFTAWRELVWPELDQLLRDEDDTTTVSTPYTAAVLEYRVEFHDPVDASLEKKSWANANGHAVIDAQHPCRANVAVRRELHTPESDRSCTHPEFDIASTGLSYETGDHVGVYCENLTEVVEEALNLLGLSPETYFSIHTDKEDGTPISGSSLPPPFPPCTLRTALAQYADLLSFPKKSSLLALAAHASNPAEADRLRHLASPAGKDEYAQWVVASQRSLIEVMAEFPSAKPPLGVFFAAVAPRLQPRYYSISSSPRMAPSRIHVTCALVYEKTPTGRIHKGVCSTWMKNAVPAEKSDDCSWAPTFVRQSNFKLPADTKVPIIMIGPGTGLAPFRGFLQERLVLKESGADLGPSILFFGCRNRRMDYIYEEELANFVETGALSELVVAFSREGPTKEYVQHKMMQKAADIWDMISQGAYIYVCGDAKGMAKDVHRTLHTIAQEQGSLDSSKAESMVKNLQMNGRYLRDVW |

**Supplementary table S3. Nucleotide sequences and protein sequence of MaC3'H.**

| **Nucleotide sequences** | >Seq1 [*Morus alba* L.] *MaC3'H*  ATGGATCTCCTTCTAATCATTCCCATCGCAATCACCCTCCTCCTTCTCTCATACAAGCTCTACCAACGGCTGAGATTCAAGCTCCCCCCGGGCCCACGCCCTTGGCCTGTAGTCGGAAACCTCTACGACATTAGGCCAGTGAGGTTCCGTTGCTTTGCGGAATGGGCTCAGTCCTATGGGCCAATCATATCGGTGTGGTTTGGGCCCACTCTGAACGTGGTGGTGTCGACGGCGGAGCTGGCGAAGGAAGTGCTGAAGGAGAATGATCAGCATCTGGCCGACCGACACCGGAGCAGATCGGCGGCGAGGTTCAGCAGAGACGGGAAGGACCTGATTTGGGCTGATTATGGACCTCATCACGTGAAGGTGAGAAAGCTTTGTACTCTTGAGCTCTTCACGCCTAAGAGGCTTGAGGCAATGAGACCCATCAGAGAGGAGGAGGTTAGGGCCATGGTTGAGTCCATTTTCAAGGACTGCGCCAATCCCGAAAACTATGGCAAAAGTTTGCGGGTGAGGGATTTCCTAGAGGCGGTGGCCTTCAACAACATAACAAGGCTGGTGTTTGGGAAGCGGTTTATGAACTCCGAGGGGGTGGTAAATGAGCAAGGGAAGGAATTCAAGGGAATTGTCTCGAACGGGATTAAGATCGGCGCGTCGCTTAACATGGCGGAGCACATCCCTTGGCTGCGTTGGGTGTTCCCACTAGAGGAGGAGGCATTCATCAAGCATGGAGATCGTAGGGACAGACTCACCAAGATCATCATGGAAGAGCACACTCAAGCTCGCAAGCAGGGTGGTGGTACCAAACAACATTTTGTCGACGCTTTGCTCACTTTGCAGGACAAGTACGACATTAGTGACGACACCATTATCGGACTCCTTTGGGACATGATCACTGCGGGCATGGACACTACCGCTATATCCGTTGAGTGGGGTATGGCTGAGCTAATCAGGAACCCGAGAGTGCAGCAAAAGGCCCAAGAAGAGCTAGACCGGGTTATTGGGTTGGAGCGGGTCCTTAATGAAACTGACTTCTCAAACCTGCCTTACCTACAATGTGTAGCTAAGGAAGCATTGCGATTGCACCCTCCAACACCATTGATGCTTCCCCACCGAGCCACCGCAAACGTCAAGATCGGCGGCTACGACATCCCTAAGGGCTCGATCGTTCACGTTAATGTCTGGGCCGTAGCCCGTGACCCAGCTGTTTGGAAAGACCCGAATGAGTTCCGCCCTGAAAGGTTCCTAGAGGAGGATGTTGACATGAAAGGCCATGATTATAGGCTTTTGCCTTTTGGGGCGGGTCGTCGGGTATGCCCCGGAGCTCAACTAGGTATAAATTTGGTTACGTCCATGTTGGGCCACCTATTGCATCACTTCCGTTGGAGTCCACCTGAAGGAATGAAGCCAGAGGACACTGACTTGGCAGAAAGCCCAGGGATGGTCACATACATGCGCACCCCATTACAAGCTATTGCCACTCCAAGGCTACCTTCCCACTTGTATAAACGCGAGGTTGTGGATGTGTAA |
| --- | --- |
| **Protein Sequence** | >Seq1 [*Morus alba* L.] MaC3'H  MDLLLIIPIAITLLLLSYKLYQRLRFKLPPGPRPWPVVGNLYDIRPVRFRCFAEWAQSYGPIISVWFGPTLNVVVSTAELAKEVLKENDQHLADRHRSRSAARFSRDGKDLIWADYGPHHVKVRKLCTLELFTPKRLEAMRPIREEEVRAMVESIFKDCANPENYGKSLRVRDFLEAVAFNNITRLVFGKRFMNSEGVVNEQGKEFKGIVSNGIKIGASLNMAEHIPWLRWVFPLEEEAFIKHGDRRDRLTKIIMEEHTQARKQGGGTKQHFVDALLTLQDKYDISDDTIIGLLWDMITAGMDTTAISVEWGMAELIRNPRVQQKAQEELDRVIGLERVLNETDFSNLPYLQCVAKEALRLHPPTPLMLPHRATANVKIGGYDIPKGSIVHVNVWAVARDPAVWKDPNEFRPERFLEEDVDMKGHDYRLLPFGAGRRVCPGAQLGINLVTSMLGHLLHHFRWSPPEGMKPEDTDLAESPGMVTYMRTPLQAIATPRLPSHLYKREVVDV |

**Supplementary table S4. PCR reaction systems of *MaCPR1* and *MaCPR2* genes**

| Reagents | Volumetric (μL) |
| --- | --- |
| cDNA | 2 |
| Primer Mix | 2 |
| dNTP Mix | 0.25 |
| Taq DNA polymerase | 0.5 |
| Green Buffer | 2.5 |
| Nuclease-free H_2_O | 17.75 |

**Supplementary table S5. PCR reaction conditions of *MaCPR1* genes**

|  | Cycle | Temperature (°C) | Time (s) |
| --- | --- | --- | --- |
| preincubation | 1 | 94 | 300 |
| 3 Step amplification | 35 | 94  57  58 | 30  30  115 |
| Extension | 1 | 72 | 420 |

**Supplementary table S6. PCR reaction conditions of *MaCPR2* genes**

|  | Cycle | Temperature (°C) | Time (s) |
| --- | --- | --- | --- |
| preincubation | 1 | 94 | 300 |
| 3 Step amplification | 35 | 94  63  58 | 30  30  115 |
| Extension | 1 | 72 | 420 |
